# Genome-Wide Survey and Expression Analysis of the KT/HAK/KUP Family in *Brassica napus* and Its Potential Roles in the Response to K^+^ Deficiency

**DOI:** 10.3390/ijms21249487

**Published:** 2020-12-13

**Authors:** Jie Zhou, Hong-Jun Zhou, Ping Chen, Lan-Lan Zhang, Jia-Tian Zhu, Peng-Feng Li, Jin Yang, Yun-Zhuo Ke, Yong-Hong Zhou, Jia-Na Li, Hai Du

**Affiliations:** 1 College of Agronomy and Biotechnology, Southwest University, Chongqing 400716, China; zj893422105@163.com (J.Z.); hsymjj@email.swu.edu.cn (H.-J.Z.); cp1996@email.swu.edu.cn (P.C.); zll0723@email.swu.edu.cn (L.-L.Z.); zhujiatian@email.swu.edu.cn (J.-T.Z.); pengfengli17@126.com (P.-F.L.); yangjin9963@163.com (J.Y.); kyz2014@email.swu.edu.cn (Y.-Z.K.); zhou2017@swu.edu.cn (Y.-H.Z.); 2 Academy of Agricultural Sciences, Southwest University, Chongqing 400716, China

**Keywords:** *Brassica napus*, potassium transporter, KT/HAK/KUP family, evolution, expression analysis

## Abstract

The KT/HAK/KUP (HAK) family is the largest potassium (K^+^) transporter family in plants, which plays key roles in K^+^ uptake and homeostasis, stress resistance, and root and embryo development. However, the HAK family has not yet been characterized in *Brassica napus*. In this study, 40 putative *B. napus* HAK genes (*BnaHAKs*) are identified and divided into four groups (Groups I–III and V) on the basis of phylogenetic analysis. Gene structure analysis revealed 10 conserved intron insertion sites across different groups. Collinearity analysis demonstrated that both allopolyploidization and small-scale duplication events contributed to the large expansion of *BnaHAKs*. Transcription factor (TF)-binding network construction, *cis*-element analysis, and microRNA prediction revealed that the expression of *BnaHAKs* is regulated by multiple factors. Analysis of RNA-sequencing data further revealed extensive expression profiles of the *BnaHAKs* in groups II, III, and V, with limited expression in group I. Compared with group I, most of the *BnaHAKs* in groups II, III, and V were more upregulated by hormone induction based on RNA-sequencing data. Reverse transcription-quantitative polymerase reaction analysis revealed that the expression of eight *BnaHAKs* of groups I and V was markedly upregulated under K^+^-deficiency treatment. Collectively, our results provide valuable information and key candidate genes for further functional studies of *BnaHAKs*.

## 1. Introduction

Potassium (K^+^) is the most abundant nutrient element in plants, accounting for 10% of the dry weight [1], and plays essential roles in protein synthesis [2], phloem transport [3], osmoregulation [4], and plant development [5]. K^+^ can also enhance the resistance to biotic and abiotic stress for improving the plant growth status. Therefore, plants often exhibit signs of chlorosis and necrosis under a state of K^+^ deficiency, resulting in a functional decline in leaf photosynthesis, and consequently compromising crop yield and quality [6,7]. The soil K^+^ concentration is generally considered to vary between 0.1 and 1 mM [8]; however, a large portion of cultivated land worldwide remains K^+^-deficient [9,10]. Consequently, the proper use of K^+^ fertilizer and increasing utilization of K^+^ will contribute to sustainable crop production and high-quality crops. *Brassica napus* is an important oil crop that requires abundant K^+^ to sustain its growth and yield, with a K^+^ demand of 290–373 kg per hectare [11,12]. However, Ren et al. [12] found that K^+^ deficiency in soil remains a long-neglected problem in China. Therefore, it is meaningful to elucidate the mechanism of K^+^ uptake in *B. napus*.

The *KT/HAK/KUP* (*HAK*) gene family encodes the most well-known high-affinity K^+^ transporters [13,14], which play important roles in diverse plant bioprocesses, especially in K^+^ uptake and transport. For example, *HvHAK1* from *Hordeum vulgare* (barley) [15], *AtHAK5* from *Arabidopsis* [16], *LeHAK5* from *Lycopersicon esculentum* (tomato) [17], and *OsHAK1* from *Oryza sativa* (rice) [18] are associated with high-affinity K^+^ uptake in the roots. Many genes such as *AtKUP7* [19], *AtKUP12* [20], *OsHAK11* and *OsHAK12* [21] are strongly induced under K^+^ deficiency. In addition, some other HAK genes (*HAKs*) have been proposed to encode low-affinity K^+^ transporters and complement K^+^ channels, such as *HvHAK2* [22] and *CnHAK1* [23]. Notably, various members of the HAK family play a crucial role in plant abiotic stress. For instance, *AtKUP6* and *OsHAK1* can enhance plant tolerance to drought [4,24], and *OsHAK16* and *ZmHAK4* can improve salt resistance [25,26]. Furthermore, *HAKs* participate in many plant growth and development processes. For example, *AtKUP9* can regulate K^+^ and auxin homeostasis to maintain root development [27]; *VvKUP1* and *VvKUP2* are likely involved in K^+^-mediated cell expansion in *Vitis vinifera* [28]; and *AtKUP2*, *AtKUP6*, and *AtKUP8* regulate K^+^ efflux to negatively affect lateral root formation [4]. Among these genes, *AtKUP2* mutants were shown to contribute to the short hypocotyl phenotype [29] and the *AtKUP4* mutant is responsible for the tiny root hair phenotype [30].

The HAK family is the largest gene family involved in K^+^ transport in plants, and has been widely identified in many plant species, including *Arabidopsis* [31], rice [14], *Triticum aestivum* (wheat) [32], and *Saccharum* (sugarcane) [33]. HAK proteins can be classified into the major facilitator superfamily of membrane transporters [34], and function as homodimers, with each monomer comprising 10–14 transmembrane (TM) domains [35,36]. In recent years, the HAK family was proposed to be classified into five groups (I–V) [37,38,39], with different numbers and attributes of *HAKs* identified in varied species or lineages.

In this study, we perform genome-wide identification and analysis of the HAK family in the *B. napus* genome, and identify 40 putative genes (*BnaHAKs*) belonging to four groups (I–III and V), with no HAKs identified in group IV. We further investigate the gene structure, chromosome localization, collinearity, transcription factors (TFs), *cis*-elements, microRNA (miRNA) targets, and protein tertiary structure for the identified *BnaHAKs*. We also analyze their expression profiles in 50 tissues at different stages throughout plant development and under five hormone treatments [abscisic acid (ABA), indoleacetic acid (IAA), gibberellin acid (GA_3_), cytokinin (6-BA), and 1-aminocyclopropanecarboxylic acid (ACC)] for 1, 3, 6, 12, and 24 h on the basis of published RNA-sequencing (RNA-Seq) datasets. Finally, the expression patterns of groups I and V *BnaHAKs* under K^+^-deficient conditions in *B. napus* seedling roots are investigated. Overall, our study provides a basis for studying the roles of *BnaHAKs* in *B. napus* in the future.

## 2. Results

### 2.1. Identification of HAK Family Members in the B. napus Genome

To identify the HAK-encoding genes in the *B. napus* genome, we performed a preliminary BLASTP search using the *Arabidopsis* HAK proteins (AtKUPs) [31] as queries. After removing the redundant and severely deleted/missing sequences, and verifying the sequences using the Pfam tool, we finally obtained a total of 40 putative *BnaHAKs* with complete functional domains. To distinguish the candidates, these genes were labeled *BnaHAK01* to *BnaHAK40* according to their chromosome locations. Using the same method, we also identified 21 and 23 non-redundant *HAKs* in *B. rapa* (*BrHAKs*) and *B. oleracea* (*BoHAKs*), respectively (Table S1). 

Physicochemical property analysis showed that the length of BnaHAK proteins (BnaHAKs) ranged from 402 to 864 amino acids. The molecular weight of the BnaHAKs ranged from 44.40 to 171.55 kDa, and the isoelectric points were concentrated at 5.22–9.44. Subcellular localization analysis showed that all 40 BnaHAKs are present in the vacuoles, and approximately half of them were also localized on the cell membrane (Table 1), which is consistent with their role in K^+^ uptake and homeostasis.

Overall, we identified a relatively larger number of HAKs in the *B. napus* genome compared with that reported for many other species [38]. This might be attributed to the fact that *B. napus* (AACC. *n* = 19) is a “young” species, which was produced by the recent hybridization between *B. rapa* (AA. *n* = 10) and *B. oleracea* (CC. *n* = 9).

### 2.2. Phylogenetic Analysis of the BnaHAK Family

To explore the evolutionary relationship of the BnaHAKs, we constructed a neighbor-joining (NJ) phylogenetic tree based on multi-sequence alignment of the 97 full-length HAK protein sequences available, including from *B. napus* (40), *B. rapa* (21) (*BrHAKs*), *B. oleracea* (23) *(BoHAKs)*, and *Arabidopsis* (13).

**Figure 1:**
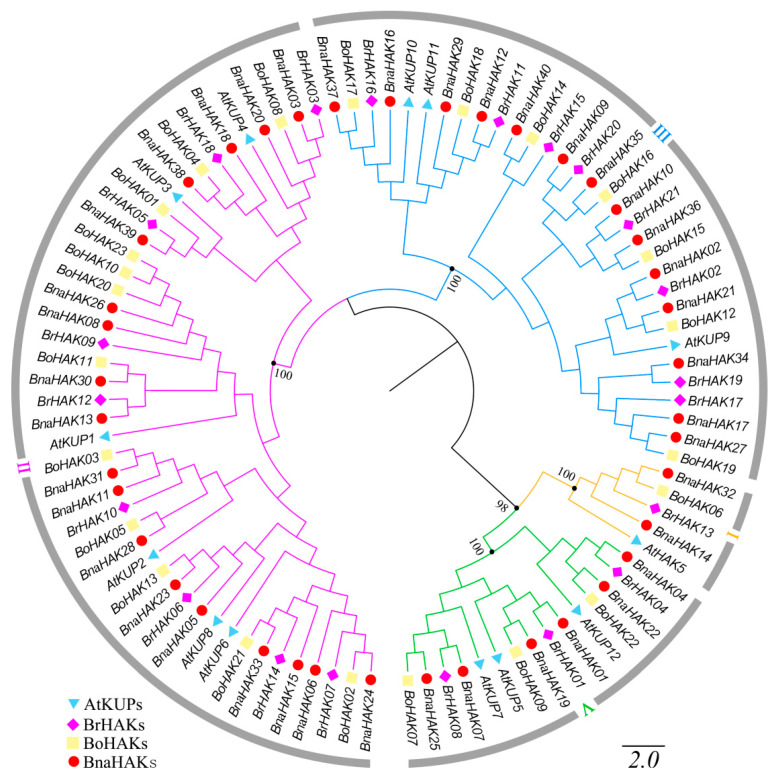
Phylogenetic analysis of KT/HAK/KUP (HAK) family proteins in *Brassica napus*, *Arabidopsis*, *Brassica rapa*, and *Brassica oleracea*. The phylogenetic tree was generated on the basis of the alignment of 97 HAK protein sequences from the four species with 1000 bootstrap replicates. The proteins belonging to the four species are represented by different shapes and colors. The HAK proteins were divided into four groups (I–III and V), which are indicated with different colored lines.

On the basis of the topology and bootstrap values of the NJ tree, HAK proteins from these four *Brassicaceae* species were classified into four groups: groups I–III and V (Figure 1). This confirmed that group IV was lost in the Brassicaceae lineage during evolution [38,39]. Group I consisted of two BnaHAKs (5%), one BrHAK, one BoHAK, and one AtKUP; group II was the largest, including 18 BnaHAKs (45%), nine BrHAKs, 12 BoHAKs, and six AtKUPs; group III had 14 BnaHAKs (35%), eight BrHAKs, seven BoHAKs, and three AtKUPs; and group V contained six BnaHAKs (15%), three BrHAKs, three BoHAKs, and three AtKUPs (Figure 1). In the phylogenetic tree, the number of BnaHAKs was double that of AtKUPs in groups I and V; however, it was three times that of AtKUPs in group II and was 4.5 times the number in group III. This suggested that the amplification rate in distinct groups varies, which might reflect the specific functional needs of each group during evolution.

### 2.3. Gene Structure and Intron Pattern of BnaHAKs

Gene structural diversity provides an important clue into the function and evolutionary history of multi-gene families. Thus, we analyzed the gene structures of the identified *BnaHAKs* using the online software GSDS 2.0.

**Figure 2:**
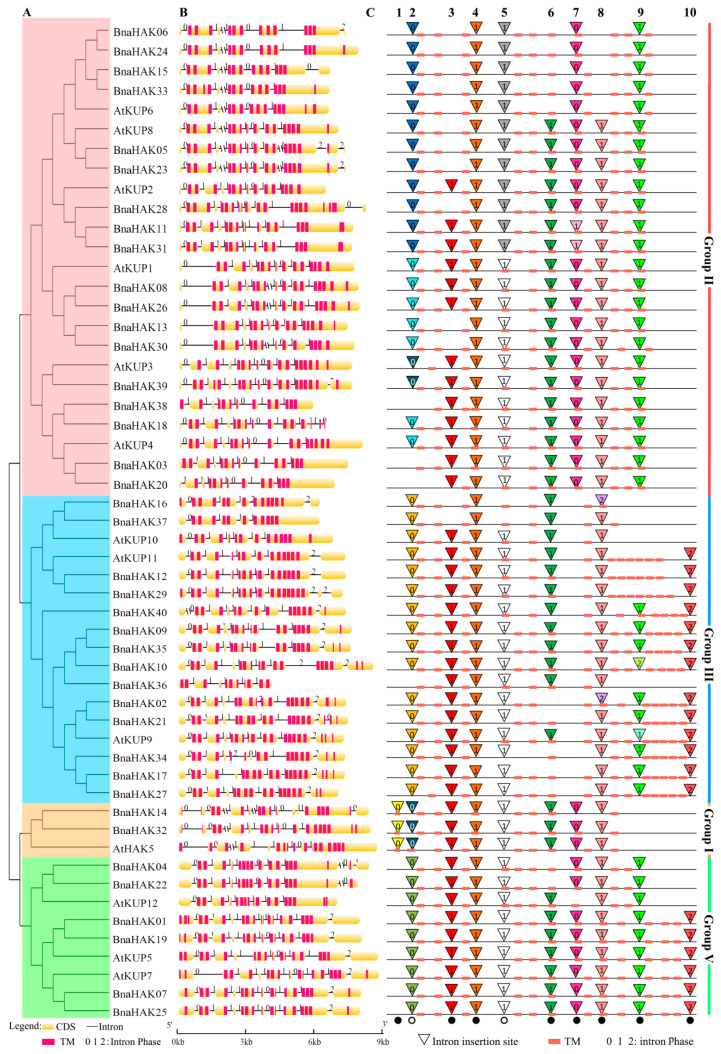
Gene structures of the candidate *HAK* genes (*HAKs*) in *B. napus* and *Arabidopsis* across different HAK family groups. (**A**) Phylogenetic analysis of *HAKs* in *B. napus* and *Arabidopsis* (*AtKUPs*). The color of the background indicates that the genes belong to different groups. (**B**) Gene structures of *BnaHAKs* and *AtKUPs*. Exons are indicated by yellow boxes, transmembrane (TM) domains are indicated by red boxes, and the spaces between the colored boxes correspond to the introns. Numbers 0, 1, and 2 represent introns in phase 0, 1, and 2, respectively. (**C**) Intron insertion patterns of *BnaHAKs* and *AtKUPs*. Each column represents an intron at the same or similar sites. Introns with the same phase and splice site are filled with the same color. The top number (1–10) is the order of the 10 introns. The black dots and black circles at the bottom indicate the highly and less conserved intron insertion sites, respectively.

The intron number varied widely among the 40 *BnaHAKs*, ranging from 4 in *BnaHAK37* to 11 in *BnaHAK18* (Figure 2B). The exon-intron structures of groups I, III, and V were relatively conserved compared with those of group II, with only a few exceptions that might have been due to the low genome sequence quality. The two *BnaHAK*s in group I contained 10 and 8 introns, respectively; the members of group III generally possessed 7–9 introns, except for *BnaHAK16*, *BnaHAK36*, and *BnaHAK37*, which contained 4 or 5 introns; group V contained 8–9 introns; and 18 *BnaHAK*s in group II contained 5–11 introns (Figure 2B). Notably, although the intron number was variable, the intron insertion sites and phase of most introns (10 introns) were conserved across the different groups (Figure 2C). Among them, intron 4 was completely conserved in all four groups; introns 3 and 6–9 were highly conserved in the four groups; and introns 1 and 10 were only conserved in groups I, and III and V respectively (Figure 2C). The insertion site of intron 2 was slightly variable, resulting in independent conserved patterns in the four groups, with three typical types found in group II (Figure 2C). Similarly, intron 5 contained two insertion types in group II. Moreover, the intron insertion patterns of *AtKUPs*, *BrHAKs*, and *BoHAKs* further validated our results in *BnaHAKs*, indicating their conservation during evolution (Figure S1). Therefore, we speculated that these conserved intron insertion sites might exist in *HAKs* of plant species. In addition, these introns were commonly inserted outside of the TM domains, except for intron 6, which was highly conserved in the same TM domain across *HAKs*. In addition, there were some unconserved intron insertion sites identified that were generally concentrated on the C-terminal (Figure S2).

Overall, these results revealed that the intron number of *BnaHAKs* differs among different groups or even within a group, but that most of the intron insertion sites are highly conserved.

### 2.4. Chromosomal Distribution and Duplication of BnaHAKs

To understand the duplication relationship of the HAK gene family in *B. napus*, we analyzed the chromosomal location and collinearity relationship of the *BnaHAKs*.

**Figure 3:**
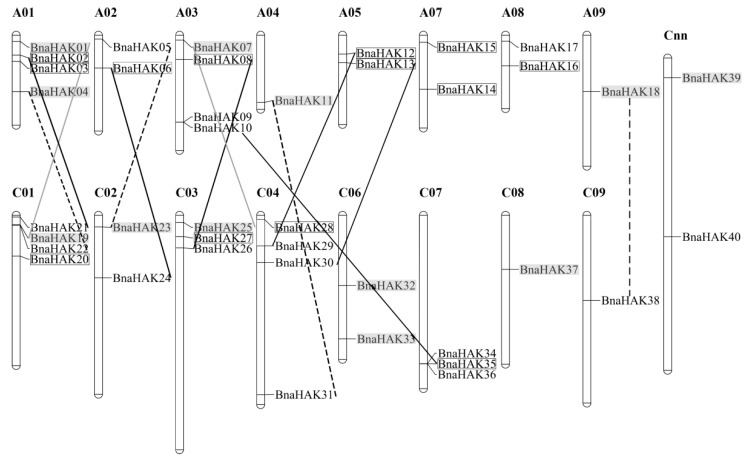
Distribution of *BnaHAKs* on *B. napus* chromosomes. The 40 *BnaHAKs* were mapped onto 16 chromosomes, except for *BnaHAK39* and *BnaHAK40* whose chromosomal locations are currently unclear. The C_nn_ chromosomes represent chromosomal fragments mapped to the C_n_ subgenome, but the detailed locations are still unclear. A black frame represents the genes that originated from a whole-genome duplication event; the grey lines and grey background represent the genes that originated from segmental exchange duplication; the black lines represent the genes that originated from homologous exchange; and the dashed lines represent the genes that originated from segmental duplication.

Our results showed that 38 of the 40 *BnaHAKs* (the detailed locations of *BnaHAK39* and *BnaHAK40* are currently unknown) were distributed unevenly across 16 of the 19 *B. napus* chromosomes, except for A_n_06, A_n_10, and C_n_05 (Figure 3). The chromosomes A_n_01, A_n_03, and C_n_01 possessed the most *BnaHAKs* (4 genes), whereas chromosomes A_n_04, A_n_09, C_n_08, and C_n_09 only had one *BnaHAK* each. Moreover, the number of *BnaHAKs* between the A_n_ (18 genes) and C_n_ (12 genes) subgenomes also appeared to be uneven (Figure 3). Collinear relationship analysis showed that 35 of the 40 *BnaHAKs* had a collinear relationship with *B. napus*, *B. rapa*, and/or *B. oleracea* homologs (Table S2). Among these 35 *BnaHAKs*, 13 (37%) were derived from whole-genome duplication (WGD), with nine genes originating from *B. rapa* and four from *B. oleracea*. The remaining *BnaHAKs* (22) originated from small-scale duplication events in *the B. napus* genome, including 12 (34%), due to segmental exchange (SE) events, six (17%) from homologous exchange (HE) events, and four (12%) from segmental duplication (SD) events. No tandem duplication (TD) events were noted in the *BnaHAKs*. Interestingly, all members in group V were derived from small-scale duplications, displaying a special evolutionary law. Moreover, most of these small-scale duplicated genes were also dated from *B. rapa*, suggesting a bias demand and/or retention for the *HAKs* from *B. rapa* in *B. napus*. The non-synonymous (Ka) to synonymous (Ks) substitution rate ratios of the collinear gene pairs were all less than 1 (Table S3), indicating that the duplicated *BnaHAKs* mainly underwent purification selection during evolution.

In summary, these results demonstrated that both a WGD event and small-scale duplication events (SE, HE, and SD) were the major driving forces for the large *HAK* expansion in *B. napus*; in particular, the *BnaHAKs* derived from *B. rapa* tended to be retained in the *B. napus* genome.

### 2.5. Regulation Mechanism in the Promoter Regions of BnaHAKs

**Figure 4:**
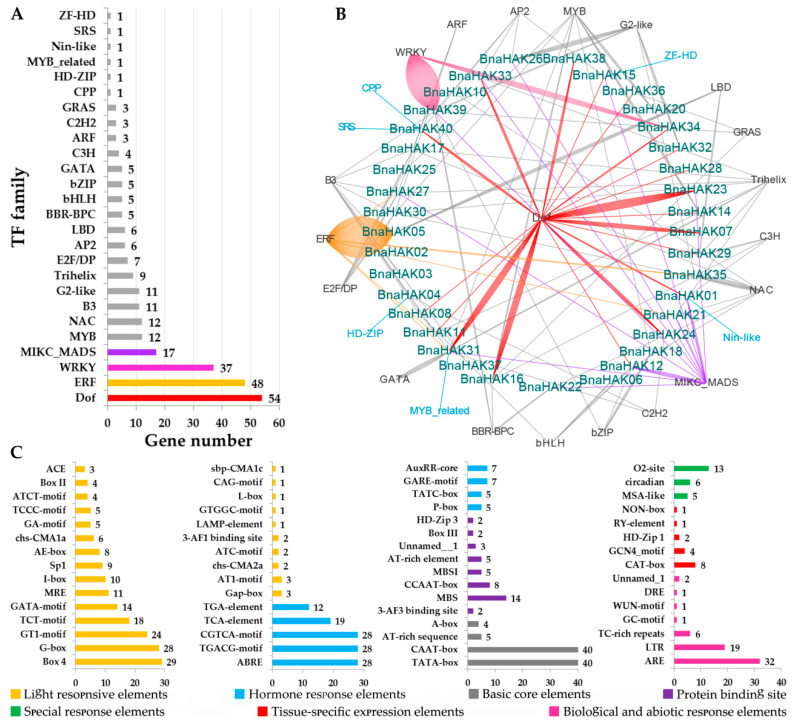
Transcription factor (TF) binding network and *cis-*element analysis in the promoter regions of the 40 *BnaHAKs*. (**A**) The TF gene families with potential binding sites in the promoter regions of 40 *BnaHAKs*. (**B**) The potential TF binding network of the *BnaHAKs* predicted using the PlantTFDB tool. (**C**) The *cis*-elements in the promoter regions of *BnaHAKs*. The abscissa represents the number of *BnaHAKs*.

We predicted the potential transcriptional regulators of the *BnaHAKs* in the PlantTFDB database using the promoter sequences (–1500 bp), and constructed a regulation network based on the results (Figure 4). Overall, we identified 269 potential TFs belonging to 26 TF families that might bind to the promoter regions of 37 *BnaHAKs* (no TF was predicted on the promoters of *BnaHAK09*, *BnaHAK13*, and *BnaHAK19*) (Table S4). The most abundant TFs belonged to the ERF (46 genes), Dof (45 genes), and WRKY (37 genes) families (Figure 4A). In addition, the Dof, MIKC_MADS, and B3 families regulated the most *BnaHAKs*, 19, 10, and 9, respectively (Figure 4B), suggesting their important roles in regulating the expression of *BnaHAKs*. The remaining TF families (including MYB, G2-like, and Trihelix) might be rare, and appear to regulate the expression of only a few *BnaHAKs*. In most cases, these TFs might bind to multiple potential *BnaHAKs* in different groups, with a few TFs only targeting a specific *BnaHAK* (Figure 4B). For instance, the ZF-HD TF might only bind to the promoter of *BnaHAK15*, SRS TF might only bind to *BnaHAK40*, and HD-ZIP TF might only bind to *BnaHAK04* (Figure 4B). 

To further explore the regulatory mechanism of *BnaHAKs*, we also analyzed the potential *cis*- elements in the promoter regions of *BnaHAKs* using PlantCARE online software. Accordingly, we identified a total of 61 types comprising 2717 *cis*-elements in the 40 *BnaHAK* promoters (Table S5). In addition to the most common basic core and light-responsive elements, we identified nine types of hormone-responsive *cis*-elements, including TGA-Element, ABRE, and P-Box, suggesting that the expression of *BnaHAKs* might be regulated by diverse hormones. Moreover, seven other types of *cis*-elements (e.g., LTRs, AREs, TC-rich repeats) were identified that are involved in biotic and abiotic responses such as low-temperature, anaerobic induction, defense and stress, and wound responses (Figure 4C).

These results showed that most *BnaHAKs* might be regulated by multiple TFs, implying a complex regulatory network for *BnaHAKs* in K^+^ uptake and transport in *B. napus*.

### 2.6. Comprehensive Analysis of miRNAs Targeting BnaHAKs

Several studies have shown that miRNAs play a significant role in targeting gene expression. Therefore, we predicted the potential miRNAs of *BnaHAKs* using the psRNATarget online software. The results indicated that 38 of the 40 *BnaHAKs* might be the targets of 145 miRNAs (no miRNA was predicted to target *BnaHAK15* and *BnaHAK27*) (Table S6), revealing the important roles of these miRNAs in *BnaHAKs* expression. The candidate miRNAs belonged to 79 families. Among them, miR2673 appears to regulate the greatest number of *BnaHAKs* (11), with the others regulating 1–7 *BnaHAKs*. Moreover, in groups II, III, and V, an individual *BnaHAK* gene could be the target of multiple miRNAs. For example, *BnaHAK05* in group II, *BnaHAK16* in group III, and *BnaHAK04* in group V might be the targets of eight, seven, and four miRNAs, respectively. Conversely, the two members in group I, *BnaHAK14* and *BnaHAK32*, might be targeted only by a single miRNA, miR8001 and miR8024, respectively. In addition, the complementarity of the miRNAs and their targeted *BnaHAKs* in the four groups were all mainly between 70% and 85% (Table S5), indicating that the sequences of miRNAs had low rates of differentiation and mutation across distinct groups during evolution. These findings provide valuable information for further elucidating the function of miRNAs in regulating *BnaHAKs* expression.

### 2.7. Characteristics of the Predicted BnaHAK Proteins

**Figure 5:**
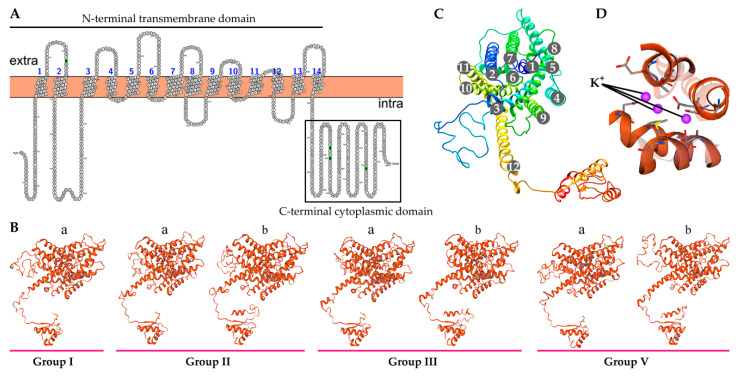
Overview of the structures of HAK proteins in *B. napus* (BnaHAKs). (**A**) Representatives of the BnaHAK tertiary structure in each group. (**B**) Topology of the BnaHAK secondary structure. (**C**) The 12 helices in the N-terminal transmembrane domain of the BnaHAKs. (**D**) The three K+-binding sites in the BnaHAKs.

We next explored the dimensional structure characteristics of the BnaHAKs, using the Protter online tool and SWISSMODEL online server to predict the secondary and tertiary structures, respectively. All 40 BnaHAKs had highly conserved secondary structures, which were commonly equipped with a conserved N-terminal TM domain and a C-terminal cytoplasmic domain, with a few exceptions that might be attributed to the deletion or lack of protein sequences (Figure 5A). Coincident with the secondary structures, the tertiary structures of BnaHAKs were also highly conserved. Significantly, there were two conserved types of the C-terminal cytoplasmic domain in groups II, III, and V identified (type “a” and type “b”), whereas the members of group I had only the type “a” domain (Figure 5B), suggesting the common origin of type “a” during evolution. In most cases, each TM domain contained 12 typical TM helices with an obvious topologically inverted repeat. Consistent with a previous study [35], these helices traversed the intra- and extracellular sections of the membranes, and the final helix extended into the cytoplasm and connected to the cytoplasmic domain (Figure 5B,C). Moreover, each TM domain generally had three consecutive K^+^-binding sites that were highly conserved in groups I and V, but 1–3 K^+^-binding site(s) were lost in groups II and III due to one or more key amino acid residue substitution(s) in these regions (Figure 5D). Considering the crucial roles of the K^+^-binding sites for BnaHAKs in K^+^ absorption, we hypothesized that these substitutions might indicate functional diversity. Furthermore, introns 3, 6, and 9 of *BnaHAKs* were conservatively distributed in the three K^+^-binding sites, implying that they might affect the function of these regions.

Overall, our results suggested that the dimensional structures of the BnaHAKs are highly conserved, and substitutions in the K^+^-binding site(s) might reflect their structural and even functional diversity during evolution.

### 2.8. Spatiotemporal Expression Profiles of BnaHAKs across Different Developmental Stages

On the basis of published RNA-Seq data (BioProject: PRJNA358784), we examined the spatio-temporal expression of 40 *BnaHAKs* in 50 *B. napus* tissues from seven organs at five developmental stages (seeding, budding, initial flowering, full-bloom, and silique stages). We excluded four *BnaHAKs* from the heatmap with no detectable transcript or weak expression levels [fragments per kilobase of exon model per million reads mapped (FPKM) < 1]. 

**Figure 6:**
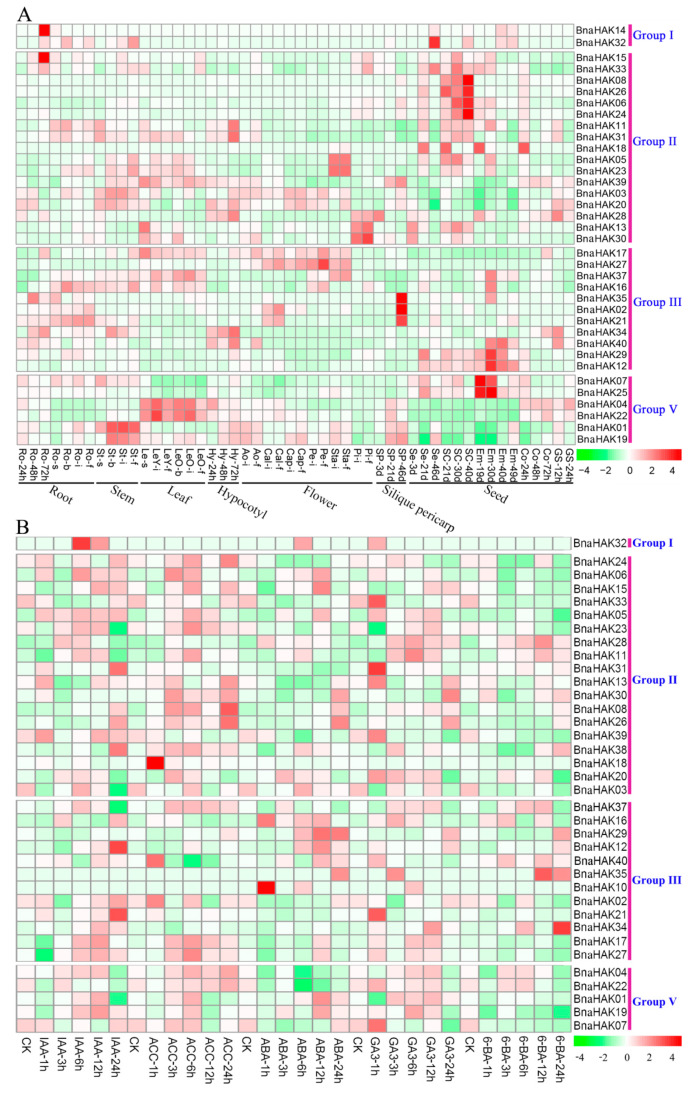
Expression pattern of 40 *BnaHAKs*. (**A**) The expression pattern of the *BnaHAKs* in 50 tissues at five developmental stages in *B. napus* determined by RNA-seq. Ro = root, St = stem, Le = leaf, Hy = hypocotyl, Ao = anthocaulus, Cal = calyx, Cap = capillament, Pe = petal, Sta = stamen, Pi = pistil, Sp = silique pericarp, Se = seed, Sc = seed coat, Em = embryo, Co = cotyledon, GS = germination seeds; “h,” “d,” “s,” “b,” “i,” “f,” and “s” indicate hour, day, seeding, budding, initial flowering, full-bloom, and silique stage, respectively. (**B**) The expression profiles of the *BnaHAKs* under five hormone treatments in *B. napus* seedling roots determined by RNA-seq. IAA: indoleacetic acid, ACC: 1-aminocyclopropanecarboxylic acid, ABA: abscisic acid, GA_3_: gibberellin acid 3, 6-BA: cytokinin. “1 h,” “3 h,” “6 h,” “12 h,” and “24 h” represent the hours after treatment. The *BnaHAKs* with no or weak expression levels (FPKM < 1) were removed from the heatmap. The color bar at the bottom side of each figure indicates the expression levels of the candidate genes.

All *BnaHAKs* showed clear spatial and temporal expression characteristics, and the expression patterns of the four groups were quite different. The expression profile of group I was relatively narrow; the two members of this group, *BnaHAK14* and *BnaHAK32*, had detectable transcript levels in the root and/or seed tissues (Figure 6A). By contrast, most of the genes in groups II and III exhibited broad expression profiles among the 50 tissues investigated (Figure 6A), indicating their wide-reaching roles in *B. napus*. The expression patterns of group V were divided into three categories, which were highly expressed in the seed, leaf, and stem tissues (Figure 6A). In general, the expression patterns of the homologs in each group were generally different, such as *BnaHAK14* and *BnaHAK32* in group I, implying their functional divergence during evolution. However, the expression profiles of sister pair genes (i.e., homologs with collinear relationships) were generally similar, with Pearson correlation coefficients greater than 0.8 (Table S7), including *BnaHAK08*/*BnaHAK26, BnaHAK12*/*BnaHAK29*, and *BnaHAK04*/*BnaHAK22* (Figure 6A), suggesting possible functional redundancy of these duplication pairs.

To explore the hormone-induced expression patterns of the 40 *BnaHAKs*, we examined their transcript levels in the seedling roots of the *B. napus* ZS11 ecotype under five exogenous hormone treatments (IAA, ACC, ABA, GA_3_, and 6-BA). Four *BnaHAKs* were excluded from the heatmap as they had no or weak expression levels (FPKM < 1). The members of group I were less sensitive to hormone treatments, as only *BnaHAK32* was induced by IAA, ABA, and GA_3_ treatments to different degrees (Figure 6B). In contrast, the expression of the majority of members in groups II, III, and V was clearly upregulated under the five hormone treatments (Figure 6B). In groups II and V, the expression of more than half of the genes was upregulated by IAA, ACC, and GA_3_ treatments (Figure 6B). In group III, the expression of most of the genes was upregulated by IAA, ABA, and GA_3_ induction (Figure 6B). These results demonstrated that most of the *BnaHAKs* in these four groups were sensitive to exogenous IAA, ACC, and GA_3_, and a few genes could also be induced by ABA and 6-BA. In addition, the sensitivity of each group to the five hormone treatments was generally divergent.

In summary, most of the 40 *BnaHAKs* showed obvious temporal and spatial as well as hormone-induced expression profiles in *B. napus*.

### 2.9. Expression Levels of BnaHAKs under Low-K^+^ Conditions 

The expression levels of *HAKs* are generally regulated by the concentration of K^+^. Among the four groups, group I is mainly involved in high-affinity K^+^ uptake, whereas most of the genes in groups II and III encode low-affinity transporters. Several genes in group V have also been related to K^+^ uptake under low-K^+^ (–K^+^) conditions [20,31]. Thus, to gain insight into the potential roles of *BnaHAKs* in response to –K^+^ stress in *B. napus* seedling roots, eight *BnaHAKs* in group I (*BnaHAK14*/*BnaHAK32*) and V (*BnaHAK07*/*BnaHAK25*, *BnaHAK04*/*BnaHAK22*, and *BnaHAK01/BnaHAK19*) were selected for reverse transcription-quantitative polymerase chain reaction (RT-qPCR) assays.

**Figure 7:**
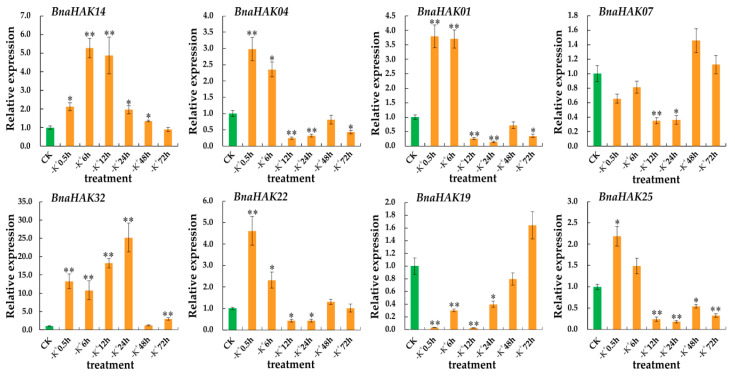
Expression levels of eight *BnaHAKs* under low-K^+^ (–K^+^) treatment determined by RT-qPCR. The relative expression levels of candidates in *B. napus* seedling roots were measured by RT-qPCR under the –K^+^ condition. CK: normal K^+^ condition; –K^+^ 0.5 h to –K^+^ 72 h: –K^+^ condition for 0.5 h–72 h. The *B. napus* Actin7 (*BnaActin7*) (GenBank accession no. AF024716) and *UBI* (*BnaUBI*) (GenBank accession no. NC027770) were used as the reference genes. The orange bars represent the expression levels of *BnaHAKs* under low-K^+^ conditions, and the green bars represent the expression levels of *BnaHAKs* under normal K^+^ conditions (CK). Error bars represent the standard deviation of three independent experiments. *: Significant difference (0.05 > *p* > 0.01); **: Extremely significant difference (*p* < 0.01).

As shown in Figure 7, the eight genes were all upregulated to varying degrees at different times under the –K^+^ condition. The expression patterns of these proteins could be divided into two main types: members of group I showed an upregulation trend at the six time points with significantly higher levels at the early stages, whereas members of group V exhibited a polarized pattern with a transient upregulation pattern under K^+^ starvation. This pattern indicated that group I might play an important role in K^+^ uptake under –K^+^ conditions. Notably, the expression profiles of the sister pairs of *BnaHAK14*/*BnaHAK32* and *BnaHAK04*/*BnaHAK22* were very similar, whereas those of *BnaHAK07*/*BnaHAK25* and *BnaHAK01/BnaHAK19* were quite different. This indicated the existence of functional differentiation of the duplicated genes during evolution in *B. napus*.

In summary, the expression of group I and V genes was sensitive to the –K^+^ environment, which provides a foundation for further exploration of their roles in K^+^ uptake in the roots.

## 3. Discussion

Given their key roles in plant K^+^ uptake, homeostasis, translocation, and stress resistance, the HAK family has been identified in many plant species such as *Arabidopsis* [31], rice [14], wheat [32], and *Zea mays* (maize) [40]. Previously, the plant HAK family was generally classified into four groups (I–IV), such as in rice and maize [14,40]. However, recent studies further divided HAKs into five groups, with the newly defined Group V separated from the old group III [38,39]. In this study, we first systematically identified the HAK family in an important oil crop, *B. napus*, and identified 40 members, which constitutes the second largest HAK family identified in plants to date [19,38]. Based on our phylogenetic, intron insertion pattern, and protein characteristic analyses, we confirmed the existence of group V, which supports this new classification of the plant HAK family. 

In previous studies, SD and TD events were reported as the major contributors to the large HAK gene expansion in many plant genomes [40,41,42,43]. However, in this study, we found that not only small-scale duplication events (including SE, 34%; HE, 17%; and SD, 12%) but also genome-wide duplication (hybridization between *B. rapa* and *B. oleracea*) were the major contributors to the rapid gene expansion of the *BnaHAK* family. This same scenario was also recently demonstrated in the sugarcane HAK family [33]. Given that Brassicaceae species have experienced a common whole-genome triplication (WGT) event, the 13 *AtKUPs* should be expanded to ~40 and ~80 genes in the *B. rapa*/*B. oleracea* and *B. napus* genomes, respectively. However, we found that 48% (19) of the *BrHAKs* and 43% (17) of the *BoHAKs* were lost after the WGT event, while most of the duplicated *BnaHAKs* were retained after the WGD event (hybridization). This might be attributed to the fact that the WGD event between *B. rapa* and *B. oleracea* occurred only about ~7500 years ago [44], which might be too short for the loss of duplicates.

Since the first *HAK* gene was cloned from barley, many homologs have been identified and functionally analyzed in various plant species (Table S8). The *HAK* genes in groups I and V were proven to be mainly involved in high-affinity K^+^ uptake and translocation. For example, *AtHAK5* (I) [16], *HvHAK1* (I) [15], *LeHAK5* (I) [17], and *AtKUP7* (V) [19] can respond to K^+^ deficiency, maintaining K^+^ uptake and transport. Similarly, we found that the expression of *BnaHAKs* in groups I and V was upregulated under the –K^+^ condition, suggesting a similar function of these genes in K^+^ uptake and transport. Interestingly, according to their expression patterns, *BnaHAK14* and *BnaHAK32* seem to have complementary functions of K^+^ uptake with each other in the roots; namely, *BnaHAK14* was highly expressed and absorbed K^+^ under control conditions, whereas the expression of *BnaHAK32* was greatly upregulated to maintain K^+^ absorption in the –K^+^ environment. 

Moreover, due to more extensive study of group I, other functions of group I members have also been identified. For instance, *AtHAK5* [45], *SIHAK5* [46], and *OsHAK1* [47] in group I can mediate high-affinity Cs^+^ uptake because of the similarity between Cs^+^ and K^+^. Some genes can also enhance plant stress resistance; for example, *OsHAK1* [24] and *HvHAK1* [48] are involved in drought stress, and *AtHAK5* [49] and *OsHAK16* [25] play a role in the salt stress response. In contrast, most members of group II are generally engaged in low-affinity K^+^ absorption and complement K^+^ channels [22,23]; the genes in group III have been shown to participate in the maintenance of K^+^/Na^+^ homeostasis, such as *AtKUP11*, *OsHAK11*, and *OsHAK12* [21]; and the genes in group IV are involved in Na^+^ uptake, such as *PpHAK13* from *Physcomitrella patens* [50], *PhaHAK5* from *Phragmites australis* [51], and *ZmHAK4* from maize [26]. Remarkably, more genes in groups II, III, and IV have been reported to be associated with various plant developmental processes. For instance, *AtKUP4* (II) is crucial for both gravitropic responses and root hair formation by mediating auxin efflux [30]; *AtKUP2*, *AtKUP6*, *AtKUP8* [4], and *CnHAK1* [23] in group II are involved in K^+^-dependent cell expansion and mediate K^+^ efflux in roots, which negatively regulate plant growth and cell size; *AtKUP4* and *OsHAK10* in group II play a role in seed maturation and reproductive processes, respectively [14,52]; *AtKUP9* in group III can maintain root growth and meristem activity by regulating K^+^ and auxin homeostasis under low-K^+^ stress [27]; the cotton *GhKT1* in group III plays a role in fiber elongation [53]; and *LjKUP* in group IV from *Lotus japonicus* is involved in nodulation development [54]. As mentioned above, group IV is absent in Brassicaceae [38]. On the basis of our findings, we suspected that this may be due to its functional redundancy with groups II and III. In summary, the five groups of the HAK family commonly play a role in plant K^+^ uptake and transport, as well as in the stress response, growth, and development. 

This study provides a useful basis for future studies on the functions of the *BnaHAKs* and can contribute to the long-term goal of improving the K^+^ utilization efficiency of oil crops.

## 4. Materials and Methods 

### 4.1. Identification and Phylogenetic Analysis of the HAK Family in B. napus

The AtKUPs were obtained from the TAIR database (http://www.arabidopsis.org). To identify the HAK-encoding genes in the *B. napus* genome, we performed a BLASTP [55] search in the GENOSCOPE database (http://www.genoscope.cns.fr/brassicanapus/), using the known AtKUPs as queries with a low-stringency criterion (cutoff *p* < 0.1). After deleting the redundant sequences, the remaining sequences were examined by the Pfam tool (http://pfam.xfam.org/search/sequence) to ensure that they contained the typical domains of the HAK family. We excluded the severely missing sequences from our dataset, and finally obtained 40 BnaHAKs. The DNA and coding sequences of the candidates were obtained from the GENOSCOPE database. Using the same method, we also identified BrHAKs and BoHAKs from the Phytozome v12.1 database (http://www.Phytozome.net) [56]. The biochemical properties of the candidates were predicted using the ExPASy tool (http://www.expasy.org/tools/) [57], and the subcellular localization was investigated using Plant-mPLoc (http://www.csbio.sjtu.edu.cn/bioinf/plant-multi/).

To explore the evolutionary relationship of the HAK family in *B. napus*, *B. oleracea*, *B. rapa*, and *Arabidopsis*, we performed multiple sequence alignment of the obtained protein sequences of BnaHAKs, BrHAKs, BoHAKs, and AtKUPs using the MAFFT version 7 tool with default parameters (https://mafft.cbrc.jp/alignment/server/). Subsequently, a phylogenetic tree was built using MEGA v5.2 with the NJ method based on the multiple sequence alignment. The parameters used in the phylogenetic analyses were as follows: Poisson correction, bootstrap with 1000 replicates, and pairwise deletion. Finally, FigTree v1.4.0 (http://tree.bio.ed.ac.uk/software/figtree/) was applied to view and edit the tree file. 

### 4.2. Gene Structure Analysis of BnaHAKs

The gene structures of *BnaHAKs* and *AtKUPs* were analyzed using Gene Structure Display Server (GSDS) 2.0 using with the DNA and coding sequences (http://gsds.cbi.pku.edu.cn/) [58]. The TM regions of *BnaHAKs* and *AtKUPs* were predicted by SMART (http://smart.embl-heidelberg.de/). By comparing the DNA and coding sequences of each *BnaHAK* using MEGA v5.2, the intron insertion sites in the corresponding protein sequences were manually located, and the intron insertion information for the *AtKUPs*, *BrHAKs*, and *BoHAKs* was acquired from Phytozome v12.1 (https://phytozome.jgi.doe.gov/pz/portal.html). 

### 4.3. Chromosomal Location and Collinearity Analysis of BnaHAKs

We acquired information on the chromosome locations of candidate *BnaHAKs* from the GENOSCOPE database. Mapchart v2.2 software was applied to draw the chromosome map of *BnaHAKs*. The cross-genome collinearity relationship of *BnaHAKs*, *BrHAKs*, *BoHAKs*, and *AtKUPs* was calculated and identified using the CoGe online tool (https://genomevolution.org/coge/). The duplication events of *BnaHAKs* were defined based on the collinearity relationship. The nucleotide substitution rate (Ka/Ks) of *BnaHAKs* was calculated using KaKs_Calculator2.0 software.

### 4.4. TF-Binding Network, Cis-Elements, and MiRNA Target Analysis

The network between *BnaHAKs* and their possible transcriptional regulators was constructed using the PlantTFDB database (http://planttfdb.cbi.pku.edu.cn/prediction.php) with the promoter region (upstream 0 to −1500 bp) of *BnaHAKs*. Only transcriptional regulators with a threshold *p*-value < 10^−6^ were retained for further analysis. Finally, the network was viewed using Cytoscape 3.6.1 software [59]. The potential *cis*-elements in the upstream promoter regions (−1500 bp) of *BnaHAKs* were predicted using PlantCARE online software (http://bioinformatics.psb.ugent.be/webtools/plantcare/html/). The potential regulatory miRNAs of *BnaHAKs* were predicted using the psRNATarget website (expectation ≤ 3) (http://plantgrn.noble.org/psRNATarget/).

### 4.5. Structure Prediction Analysis of BnaHAK Proteins

To explore the tertiary structure features, the protein sequences of BnaHAKs were submitted to SWISS-MODEL, a protein-modeling server (https://swissmodel.expasy.org/interactive). To validate the secondary structural information, further analysis was performed by submitting the protein sequences of *BnaHAKs* to the Protter v1.0 online tool (http://wlab.ethz.ch/protter/) [60].

### 4.6. Gene Expression Analysis

We used an RNA-Seq dataset in the National Center of Biotechnology Information (NCBI) (BioProject: PRJNA358784) to detect the temporal and spatial expression patterns of *BnaHAKs* across all developmental stages of the *B. napus* cultivar ‘Zhongshuang 11′ (ZS11). Similarly, an RNA-Seq dataset of *B. napus* ZS11 seedling roots under five hormone inductions (IAA, GA3, 6-BA, ABA, and ACC) was obtained from NCBI (BioProject ID PRJNA608211) to explore the hormone-responsive expression patterns of *BnaHAKs*. The R package v3.5.3 [61] was used to analyze and draw a heatmap based on log2-transformed data. Genes with FPKM < 1 might be pseudogenes or only expressed under specific conditions; thus, they were excluded from the heatmap. The Pearson correlation coefficient was obtained by calculating the expression levels of homologous genes in different tissues/organs.

### 4.7. Plant Materials and Growth Condition

Seeds of ZS11 were obtained from the College of Agriculture and Biotechnology, Southwest University. The seeds were germinated in individual plastic pots filled with vermiculite, grown in an artificial climatic chamber at 25 °C with a 16:8 h photoperiod (day:night), and watered with 1/2-strength Hoagland solution every four days. Then, seedlings at the four-leaf stage were changed from soil culture to hydroponic culture with 1/2-strength Hoagland solution. The solutions were changed every three days. The seedlings at the five-leaf stage were used for the –K^+^ treatment. In this treatment, three independent repeated trials were performed, each with three plants harvested at each sampling. The normal nutrient solution comprised 1.25 mM KNO_3_, 1.5 mM Ca(NO_3_)_2_, 0.75 mM MgSO_4_, 0.5 mM KH_2_PO_4_, 75 µM FeEDTA, 50 µM H_3_BO_3_, 10 µM MnCl_2_, 2 µM ZnSO_4_, 1.5 µM CuSO_4_, and 0.075 µM (NH_4_)_6_Mo_7_O_24_ (control, CK), whereas for the –K^+^ treatment, 1.25 mM KNO_3_ and 0.5 mM KH_2_PO_4_ were replaced by 0.5 mM phosphoric acid [31]. The pH was adjusted to 5.8 with Tris. The root tissues were harvested at 0.5, 6, 12, 24, 48, and 72 h after the treatments, immediately frozen in liquid nitrogen, and then stored at −80 °C for RNA isolation.

### 4.8. RT-qPCR Analysis of BnaHAKs under Low-K^+^ Conditions

The EASYspin total RNA Extraction kit (Biomed, Beijing, China) was used to extract the total RNA from each sample. The concentration and quality of the total RNA were tested using gel electrophoresis and a NanoDrop 2000 spectrophotometer to confirm that the A260/280 ratio remained at 1.8–2.1, and that the A260/230 ratio exceeded 2.0. The RNA sample was treated with DNase I (Promega, Beijing, China), and was then used for cDNA synthesis by reverse transcription in a 20-µL reaction system according to the manufacturer’s instructions of the M-MuLV RT kit (Takara Biotechnology, Beijing, China). The primers used in this experiment were designed using Primer Premier 5 software and are listed in Table S9. *BnaActin7* (GenBank accession no. AF024716) and *BnaUBI* (GenBank accession no. NC027770) served as double reference genes. The SYBR-Green PrimeScript RT-PCR Kit (Takara Biotechnology, Beijing, China) was used for real-time PCR analysis using the CFX Connect™ Real-Time System (Bio-Rad, Chongqing, China), and every reaction system consisted of three technical replicates. The thermocycling parameters included initial denaturation at 95 °C for 5 min, followed by 45 cycles of denaturation at 95 °C for 15 s and annealing at 60 °C for 15 s (the annealing temperature of *BnaHAK32* and *BnaHAK19* was 55 °C). Finally, we obtained the data (mean ± standard deviation) of all three independent repeated trials and calculated the relative expression of *BnaHAKs* using the 2^(−∆∆Ct)^ method. Error bars represent standard errors from three independent repeated trials. Differences in expression levels in *BnaHAKs* according to K^+^ treatments were assessed by one-way analysis of variance (* *p* < 0.05; ** *p* < 0.01) using Excel 2010.

## Figures and Tables

**Table 1:** Features of the 40 KT/HAK/KUP genes (*BnaHAK*s) identified in *Brassica napus*.

Gene Name	Genome ID	Chromosome	Protein Length (aa)	CDS Length	DNA Length	pI	Molecular Weight	Subcellular Localization
*BnaHAK01*	BnaA01g03380D	chrA01	856	2571	3630	5.22	94.66	Vacuole
*BnaHAK02*	BnaA01g10310D	chrA01	812	2439	3238	6.78	90.71	Vacuole
*BnaHAK03*	BnaA01g13320D	chrA01	782	2349	3779	9.37	87.82	Cell membrane, Vacuole
*BnaHAK04*	BnaA01g22220D	chrA01	834	2868	5005	5.61	106.49	Cell membrane, Vacuole
*BnaHAK05*	BnaA02g02340D	chrA02	710	4299	8919	8.48	161.23	Vacuole
*BnaHAK06*	BnaA02g14820D	chrA02	665	1998	3831	8.75	74.33	Cell membrane, Vacuole
*BnaHAK07*	BnaA03g02700D	chrA03	864	2595	3806	5.30	96.22	Vacuole
*BnaHAK08*	BnaA03g13690D	chrA03	711	2136	5533	8.03	78.97	Cell membrane, Vacuole
*BnaHAK09*	BnaA03g44320D	chrA03	808	2427	3476	7.04	91.09	Vacuole
*BnaHAK10*	BnaA03g44330D	chrA03	810	2433	3761	6.28	91.26	Cell membrane, Vacuole
*BnaHAK11*	BnaA04g23330D	chrA04	776	2331	3847	6.97	87.06	Vacuole
*BnaHAK12*	BnaA05g08850D	chrA05	787	2364	3752	8.65	88.09	Vacuole
*BnaHAK13*	BnaA05g12300D	chrA05	714	2145	3338	7.54	79.24	Cell membrane, Vacuole
*BnaHAK14*	BnaA07g16500D	chrA07	747	2244	7046	8.00	84.14	Cell membrane, Vacuole
*BnaHAK15*	BnaA07g38760D	chrA07	700	2103	3392	8.86	78.23	Cell membrane, Vacuole
*BnaHAK16*	BnaA08g08020D	chrA08	757	2274	2724	8.38	85.19	Cell membrane, Vacuole
*BnaHAK17*	BnaA08g30510D	chrA08	808	2427	3259	6.61	90.57	Vacuole
*BnaHAK18*	BnaA09g21950D	chrA09	489	1476	2821	9.25	55.07	Cell membrane, Vacuole
*BnaHAK19*	BnaC01g04660D	chrC01	850	2553	3831	5.27	94.31	Vacuole
*BnaHAK20*	BnaC01g15360D	chrC01	705	2118	3625	9.34	78.82	Cell membrane, Vacuole
*BnaHAK21*	BnaC01g41320D	chrC01	789	2373	3584	7.59	88.43	Vacuole
*BnaHAK22*	BnaC01g43090D	chrC01	832	2700	4041	5.75	99.91	Cell membrane, Vacuole
*BnaHAK23*	BnaC02g05800D	chrC02	775	4581	10,988	8.37	171.55	Vacuole
*BnaHAK24*	BnaC02g19780D	chrC02	777	2334	4163	8.68	86.67	Vacuole
*BnaHAK25*	BnaC03g03790D	chrC03	864	2595	3755	5.30	96.21	Vacuole
*BnaHAK26*	BnaC03g16580D	chrC03	710	2133	3984	8.21	78.89	Vacuole
*BnaHAK27*	BnaC03g76940D	chrC03	804	2415	3188	6.89	90.16	Vacuole
*BnaHAK28*	BnaC04g01430D	chrC04	784	3825	6298	6.23	142.58	Vacuole
*BnaHAK29*	BnaC04g10260D	chrC04	728	2187	3978	9.03	81.62	Cell membrane, Vacuole
*BnaHAK30*	BnaC04g14750D	chrC04	712	2139	4392	7.81	79.02	Cell membrane, Vacuole
*BnaHAK31*	BnaC04g47240D	chrC04	777	2334	3898	6.83	87.16	Vacuole
*BnaHAK32*	BnaC06g15440D	chrC06	784	2355	8981	7.64	88.06	Cell membrane, Vacuole
*BnaHAK33*	BnaC06g31400D	chrC06	785	2358	3467	8.47	87.48	Vacuole
*BnaHAK34*	BnaC07g36080D	chrC07	709	2130	3221	6.61	79.36	Cell membrane, Vacuole
*BnaHAK35*	BnaC07g36130D	chrC07	798	2397	3462	6.51	89.55	Vacuole
*BnaHAK36*	BnaC07g36140D	chrC07	402	1209	2867	8.98	44.40	Vacuole
*BnaHAK37*	BnaC08g09300D	chrC08	794	2385	3563	8.65	89.18	Vacuole
*BnaHAK38*	BnaC09g24170D	chrC09	539	1620	5134	9.44	75.90	Vacuole
*BnaHAK39*	BnaCnng05490D	chrCnn_random	770	2313	3546	9.19	85.60	Vacuole
*BnaHAK40*	BnaCnng46720D	chrCnn_random	794	2385	4156	8.53	88.49	Cell membrane, Vacuole

Abbreviations: aa, amino acids; CDS, coding sequence; pI, isoelectric point.

## Supplementary Materials

The following are available online at https://www.mdpi.com/1422-0067/21/24/9487/s1.
