# Supplementary material for: Genome-Wide Survey and Expression Analysis of the KT/HAK/KUP Family in Brassica napus and Its Potential Roles in the Response to K+ Deficiency
Source: Int J Mol Sci. 2020 Dec 13;21(24):9487. doi: 10.3390/ijms21249487 (PMC7763660; doi:10.3390/ijms21249487)
Supplement: Supplementary file 1 [file ijms-21-09487-s001.zip › PAPER 5 Supporting information-Final.docx]

**Supporting information: Maternal responses and adaptive changes to environmental stress via chronic nanomaterial exposure: differences in inter and transgenerational interclonal broods of *Daphnia magna***

**1 Methods**

**1.1 Media and representative waters**

Commercially available analytical reagent grade chemicals were purchased from Sigma-Aldrich (Dorset, UK). Ultrapure water (UPW) with a maximum resistivity of 18.2 M Ω cm^−1^ was used in all experiments. All experiments were performed using *Daphnia* high hardness combo medium (HH combo) (Kilham et al., 1998). This medium represents a hard water standard without any natural organic matter (NOM) and is commonly used for the culturing and maintenance of *Daphnia*. A description of the chemicals that make up HH combo and the various vitamin stocks for *Daphnia* maintenance are described in the following tables.

| **Table SI.1: HH combo Media composition** | |
| --- | --- |
| **Compound** | **Stock (g L^-1^)** |
| [Calcium Chloride, Dihydrate](http://avogadro.chem.iastate.edu/msds/cacl2-2h2o.htm) | 110.28 |
| Magnesium sulphate heptahydrate | 113.5 |
| Potassium phosphate dibasic | 1.742 |
| Sodium nitrate | 17 |
| Sodium metasilicate nonahydrate | 28.42 |
| Boric acid | 24 |
| Potassium chloride | 5.96 |
| [Sodium Bicarbonate](http://avogadro.chem.iastate.edu/msds/nahco3.htm) | 63 |
| **pH** | 7.6-7.8 |
| **Ionic strength without animate** | 11.07 |
| **Ionic strength with animate** | 20.83 |

| **Table SI.1A: Animate** | |
| --- | --- |
| **Compound** | **Stock (g/100mL)** |
| lithium chloride | 31 |
| rubidium chloride | 7 |
| strontium chloride hexahydrate | 15 |
| sodium bromide | 1.6 |
| potassium iodide | 0.33 |

| **Table SI.1B: Vitamin stock solution** | |
| --- | --- |
| **Compound** | **Stock** |
| Biotin | 10 mg/96 mL |
| B_12_ | 10 mg/89 mL |
| Thiamine HCl | 10 mg/50 mL |

To prepare 1L stock solution of animate, 1mL of each compounds reported in Table SI.1A is added. From the 1L stock solution of animate, 1 mL is added to every 1 L of the HH combo medium. The vitamin stocks are prepared using an aliquot of 1.5 mL of biotin and B_12_ in 50 mL total volume MQ water, with 10 mg of Thiamine HCl added (Table SI.1B). A total of 0.5mL from the vitamin stocks are added to every 1 L of HH combo.

**1.2 Range-finding study (Daphnia acute immobilization test)**

When selecting test concentrations for NMs toxicity testing, there is always a trade-off between utilising environmentally realistic concentrations, and effective concentrations (ECs) at which some effect from the test material is observed. Our justification for using ECs in this study is because regulation and environmental risks are assessed by characterizing the effects in biological receptors. Furthermore, the concentrations ranges at which toxicity has been observed for various TiO_2_ and Ag NMs have been reported elsewhere (Kim et al., 2010, Zhu, 2010). To confirm that our particles behave in a broadly similar manner, a range finding study was undertaken to determine the EC_50,_ using acute 48 hour immobilization tests. The results are shown in Figure SI.1. The pristine uncoated TiO_2_ were overall less toxic compared to the PVP coated TiO_2_ NMs, particularly at higher concentrations (Figure SI.1A) and as expected the Ag_2_S NMs were much less toxic than the uncoated Ag NMs (Figure SI.1B). Using the range finding curves (Figure SI.1), EC_30_ values were established for use in the pilot 28-day studies, using exposure concentrations of 45mg L^-1^ (TiO_2_ PVP), 30 mg L^-1^ (TIO_2_ uncoated), 20 µg L^-1^ (PVP Ag), 20 µg L^-1^ (uncoated Ag) and 100 µg L^-1^ (Ag_2_S NMs), respectively. The pilot studies were conducted to identify any issues with the study design and/or the NM concentrations used. The EC_30_ concentration from the acute studies showed high mortality in the TiO_2_ NM pilot studies with almost 100% mortality after 6 days of exposure for those exposed to pristine uncoated TiO_2,_ possibly due to the effects of feeding. For this reason the test concentrations for the multi-generational studies were reduced further to the EC_5_ values of 5 mg L^-1^ for the TiO_2_ NMs and, which matched the exposure concentrations reported in other *Daphnia* toxicity studies utilising TiO_2_ NMs (Kim et al., 2014). No adjustments were required for the Ag NM studies.

**
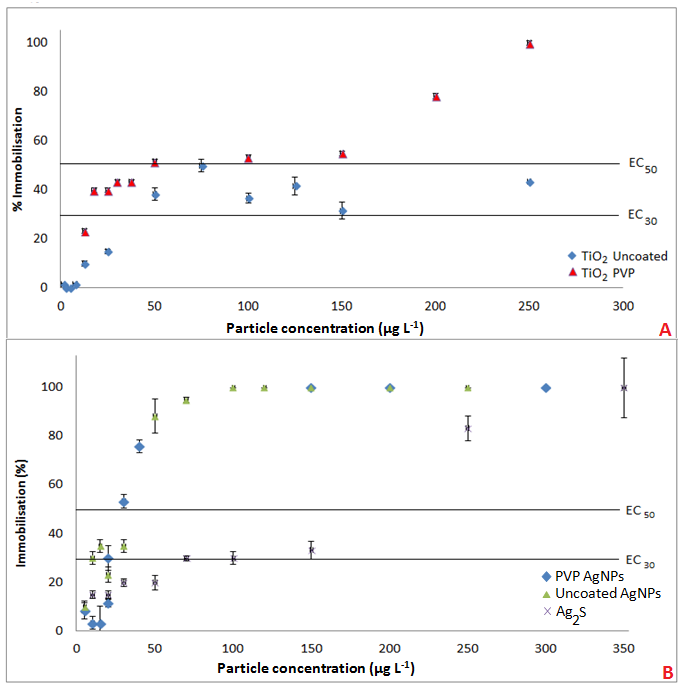
**

**Figure SI.1A:** EC_50_ range finding concentrations Vs immobilization (%) for *Daphnia* exposed to the differently coated TiO_2_ NMs over 48 hours and **1B:** range finding concentrations Vs immobilization (%) for *Daphnia* exposed to the differently coated Ag NMs.

- 1. **Gene expression**

**1.3.1 RNA extraction**

After exposure (or removal- depending on the generation) to the NMs for 24 hours, a total of 20 neonates (≤ 48 hours old) from each sample generation were euthanized using liquid nitrogen and stored in precellys tubes (with approximately 30 beads) at -80^o^C until extraction. An Agencourt RNAdvance Tissue Kit (Beckman Coulter A47943) using paramagnetic bead-based technology was used for total RNA isolation and purification. Firstly, purification of whole *D. magna* samples (as per the manufacture’s recommended protocol), involved the preparation of 20 µL of proteinase K with 400 µL lysis buffer (per sample), followed by mechanical homogenization using a Precellys 24 instrument (Bertin Technologies) using 1 cycle of 20 seconds at a 6400 pulse speed. The samples were then incubated at 37^o^C for 25 minutes and transferred to a 96 well plate.

To isolate the RNA in the samples, a Beckman Coulter Biomek FxP was used to automate the immobilization of the RNA onto magnetic particles separating it from any other contaminants in the samples. This was completed by adding 400 µL bind buffer (containing 80 µL of bind buffer and magnetic beads with 320 µL of isopropanol) to each sample, whilst shaking to mix thoroughly for 5 minutes. The plate was placed on the magnet for 6 minutes to separate the beads from the mixture, where the supernatant was then removed from each sample. The plate was then removed from the magnet and the samples are washed with 70% ethanol, covered with plate seal and stored at -80^o^C until required. RNA yield was quantitated by use of a NanoDrop ND-8000 (ThermoFisher ND-8000-GL). Aliquots of each sample were diluted to ~5ng per µL, and tested upon the Agilent Tapestation 2200 (Agilent G2964AA) with High Sensitivity RNA screen tapes (Agilent 5067- 5579) to ascertain the RNA Integrity Number.

- - 1. **Preamplification/Reverse transcription**

A total of 8 genes were selected for target-specific amplification using a mix of previously published primer sequences (Table SI.2). Primer sequences were also checked using NCBI primer blast software (<https://www.ncbi.nlm.nih.gov/gene>) for the probability of amplifying nonspecific products. A Onestep qPCR kit (Qiagen) was used in accordance to the manufactures guidance. Briefly, 800ng of each sample was combined with primers for each gene (from a 100µM stock) and added to 10µl buffer, 2µL dNTP mix, and 2µL enzyme mix. Water was added to bring the final volume to 20µL. Reverse transcription was facilitated by a 30 minute incubation at 50^0^C. Following reverse transcription the samples were used to setup 2 separate pre-amplification plates (due to differences in primer annealing temperatures) (Table SI.3).

Gene assay mixtures were created by mixing 1µLof a 100µM stock of each primer. Set A consisted of GST, NADH and HO1 (note that 18s did not undergo pre-amplification due to its high level of expression), set B consisted of β-actin, DNA Polymerase, catalase, and Metallothioenen. Gene assay mixtures were then diluted adding sufficient DNA suspension buffer (TEKnova T0221) to bring the volume to 200 µL. For each sample 1.25 µl cDNA was transferred to a clean plate. To this 1 µL Pre-amp master mix (Fluidigm 100-5580), 0.5 µL Pooled Gene assay mix, and 2.25µL DNase-free water was added. The plate was placed in a Eppendorf Mastercycler nexus gradient (Eppendorf 6331000017) on the protocol outlined in Table SI.3. Following pre-amplification the reaction mixtures (Table SI.4) were cleaned up using Exonuclease I (Table SI.5). To each sample the following was added; 1.4 µl DNAse free water, 0.2 µl ExoI reaction buffer, and 0.4 µl ExoI (NEB M0293L). Samples were mixed, and thermal cycled at the following conditions;

1. 37^0^C for 30 mins
2. 80^0^C for 15 mins
3. Held 4^0^C (until sample removed from cycler)

After ExoI treatment the samples were diluted with 25 µl DNA suspension buffer (TEKnova T0221).

Diluted samples were stored at -20^o^C until ready for Fluidigm Gene Expression.

The Preamp Gene Assay Master Mix was combined to produce the preamplification pre-mix (Table SI.4) which is individually separated into inlets of a 96 well sample plate. Each individual sample (measured in triplicate) containing the cDNA is added into one of the well inlets (1 inlet per sample). The preamplification was conducted on an Eppendorf Mastercycler Nexus eco gradient model with a 2 minute hold at 95^o^C, 50^o^C for 30 minutes and held at 4^o^C. RNA integrity was measured using an Agelent Technologies 2200 TapeStation. Specific target amplification products were then treated Exonuclease I (Exo I) (New England BioLabs) to degrade any unbound primers (Table SI.5).

| **Table SI.2: Target genes; primer sequence, function and reference** | | | | |
| --- | --- | --- | --- | --- |
| **Target gene** | **Forward Primer 5'-3'** | **Reverse Primer 3'-5'** | **Function** | **Reference** |
| **Glutathione s-transferase (GST)** | CAA CGC GTA TGG CAA AGA TG | CTA GAC CGA AAC GGT GGT AAA | xenobiotic detoxification, oxidative stress | (Qiu et al., 2015) |
|  |  |  |  |  |
| **Dehydrogenase (NADH)** | GCA GGA AAC AAT AAG GCA AAC C | GGT GGC ACA GAC CAT TTC TTA | Mitochondrial electron transport and energy production | (Qiu et al., 2015) |
| **β-Actin (B-Actin)** | CCA CAC TGT CCC CAT TTA TGA A | CGC GAC CAG CCA AAT CC | Reference gene: cytoskeleton production and cell maintenance (house keeping gene) | (Qiu et al., 2015) |
| **Catalase (CAT)** | CAG GAT CAT CGG CAG TTA GTT | CTG AAG GCA AAC CTG TCT ACT | Oxidative stress attenuation | (Qiu et al., 2015) |
| **Metallothionein (MET)** | GTGGAACCGAATGCAAATG | TGCATGGACAACTGGAACTG | Metal/ xenobiotic detoxification | (Poynton et al., 2007) |
| **DNA Polymerase (DNA-poly)** | ATGCGTTAGGCGTCAATACC | TACAAGGTTTGCCCTTGCTT | DNA repair | (Poynton et al., 2007) |
| **18S ribosomal RNA (18S)** | CGC TCT GAA TCA AGG GTG TT | TGT CCG ACC GTG AAG AGA GT | Reference gene: Protein biosynthesis (reference gene) | (Heckmann et al., 2006) |
|  |  |  |  |  |
| **Heme-oxygenase-1 (HO1)** | TATGGAGCACAATGGCTTGA | GGATTTGACCTCCCGAAAAT | Metal detoxification | GenbankEFX79040.1 |

| **Table SI.3 pre amplification** | |
| --- | --- |
| **Cycle number** | **Condition** |
| 1 | 95^0^C for 3 minutes |
| 12 (or 16 for low input samples) | 95^0^C for 5 secs |
|  | 50^0^C or 52^0^C for 30 secs |
|  | 60^0^C for 4 minutes |
| Held (until sample removed) | 4^0^C |

| **Table SI.4: Preamplification pre-mix** | |
| --- | --- |
| **Contents** | **Volume per well inlet (µL)** |
| Preamp Master Mix | 1 |
| Gene Assay Mix (500 nM) | 0.5 |
| Dnase-free water | 2.25 |
| cDNA sample | 1.25 |
| **Total** | **5** |

| **Table SI.5: Exonuclease I treatment** | |
| --- | --- |
| **Contents** | **Volume per well inlet (µL)** |
| Dnase-free water | 1.4 |
| Exonuclease I Reaction+ Buffer | 0.2 |
| Exonuclease I, 20 u/µL | 0.4 |
| **Total** | **2** |

**1.3.3 qPCR**

Gene expression was conducted using Flex Six Integrated Fluidic Circuit (IFC) Delta Gene Assay (72 x 72) in combination with a HX Prime (153x) system and a Fluidigm BioMark (Standard) Real time PCR instrument, as per the manufacturer’s recommended protocol. The purified Exo I treated samples were mixed with the EvaGreen supermix and 2x loading assay (Table SI.6) to produce the sample pre mix. The samples were then utilized for high-throughput qPCR on 72 independent samples across 72 qPCR assay probes, equivalent to 5184 independent reactions. The IFC Delta Gene Assay partitions the sample into 72 microfluidic chambers and performs qPCR detection and quantification for each specific gene in each chamber. For each Dynamic Array used, we enriched each sample and gene in triplicate using a 12 x 12 format to utilise the 72 assay chambers.

The Flex Six IFC was primed with 150 µL of the control line fluid for 15 minutes prior to loading the samples, using the prime script (153x) feature on the HX instrument (BioMark, Fluidigm). Samples and gene assays were loaded into the IFC and the ‘run script’ on the HX instrument enabled the loading of the samples and assays into the camber for 50 minutes before being run on the Biomark instrument according to conditions outlined in Table SI.7.

| **Table SI.6: Assay and Sample Pre-Mixes** | | | |
| --- | --- | --- | --- |
| **Gene Assay mix** | | **Sample Pre-Mix** | |
| **Component** | **Volume per well inlet (µL)** | **Component** | **Volume per well inlet (µL)** |
| 100 M of forward and reverse primers | 0.15 | SsoFast EvaGreen Supermix with Low ROX | 1.5 |
| DNA Suspension Buffer | 1.35 | Flex Six Delta Gene Sample Reagent | 0.15 |
| 2X Assay Loading Reagent | 1.5 | PreAmp and Exo I treated sample (to be added individually) | 1.35 |
| **Total** | **3** | **Total** | **3** |

| **Table SI.7: qPCR conditions** | | | |
| --- | --- | --- | --- |
| **Conditions** | **Cycle number** | **Temperature** | **Time** |
| Hot start | 1 | 95^o^C | 3 minutes |
| Amplification | 40 | 95^o^C | 5 seconds |
|  |  | 50^o^C (Set A) or 52^o^C (set B) | 30 seconds |
| Melt | 1 | 95^o^C | 30 seconds |
|  |  | 50^o^C | 30 seconds |
|  |  | 95^o^C | 30 seconds |

**2 Results**

**2.1 Nanomaterial preparations and characterization**

It is important to note that TEM only measures a very small fraction of the sample, and in order to be able to size the individual particles it is necessary to focus on areas that are not so highly aggregated. While the TEM images (Figure SI.2) clearly show aggregation between the different sets of particles, in many cases the aggregates were so large it was impossible to fit them into a single image to allow for sizing. For this reason, only the individual (primary) particle sizes are reported from the TEM data. The DLS reports the z-average size, which by definition, is an average over all sizes detected and as scattering scales 1/D^6^ (where D is the particle diameter) it skews the size towards larger particles. Thus, the absolute numbers are not the main message from Table I, but rather changes in the numbers as a result of the ageing processes in the different media are the important feature (Ellis et al., 2020).

Uncoated TiO_2_ PVP TiO_2_ Uncoated Ag PVP Ag Ag_2_S

**
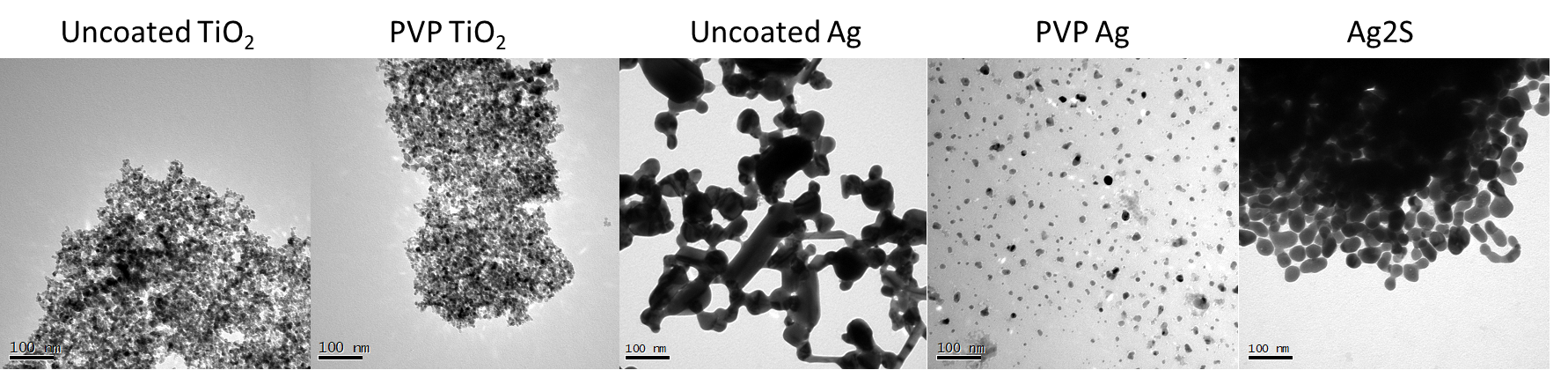
**

**Figure SI. 2. TEM images of: A**: Uncoated TiO_2_, **B:** PVP TiO_2_, **C:** Ag_2_S, **D:** PVP Ag, **E:** Uncoated Ag NMs.

**2.2 Longevity**

**
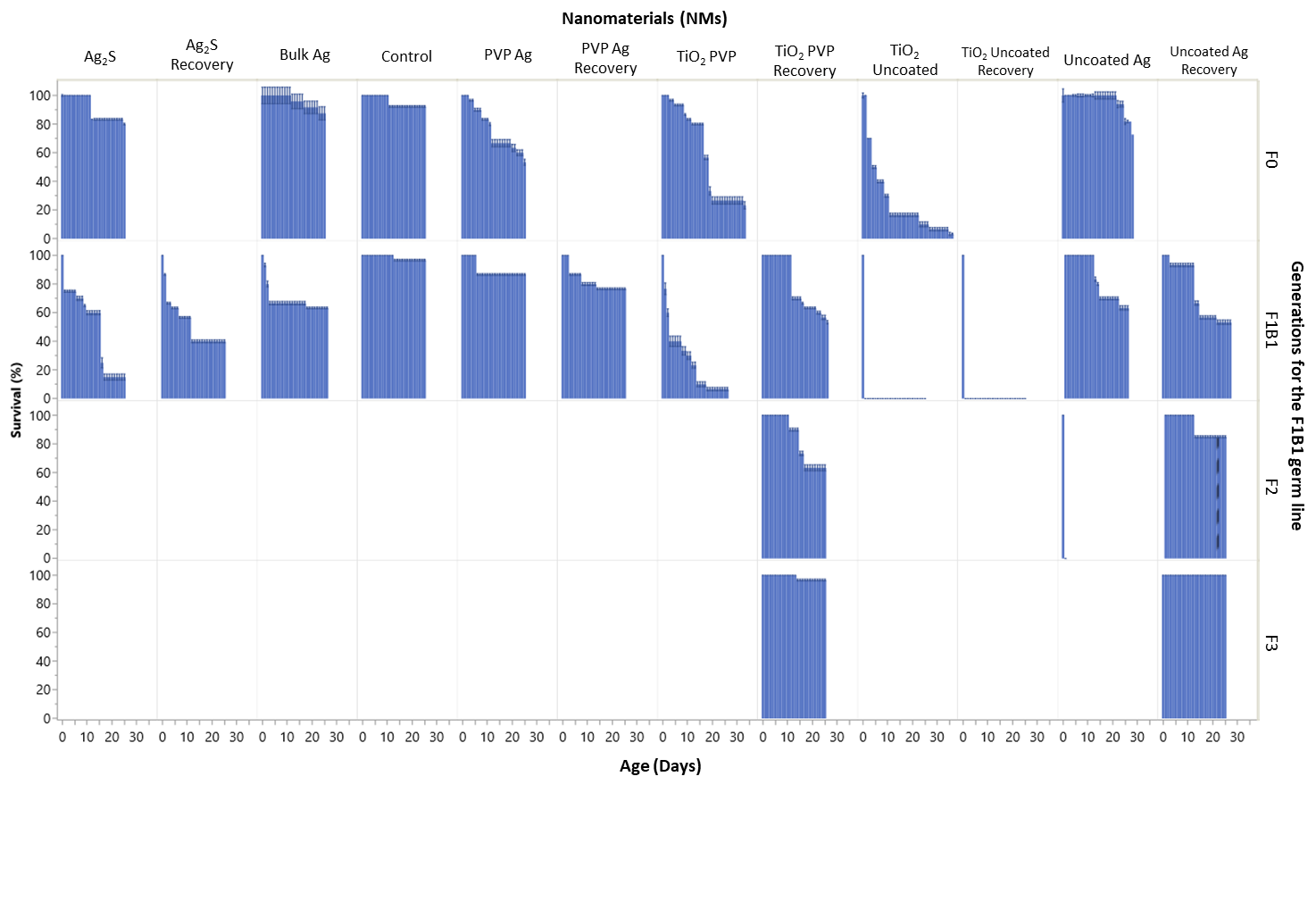
**

**Figure SI.3:** Multigenerational longevity for daphnids both continuously exposed to each of the Ag and TiO_2_ NMs, and those in the recovery, for the subsequent generations of the F1B1 germ lines. The Y-axis indicates the average survival (%) versus the *Daphnia* age (in days at the time of measurement) on the X-axis. F0 = Parent exposure to the particular NM is noted at the top of the plots, and the following plots are split for each generation. Data are mean ± SD.

**
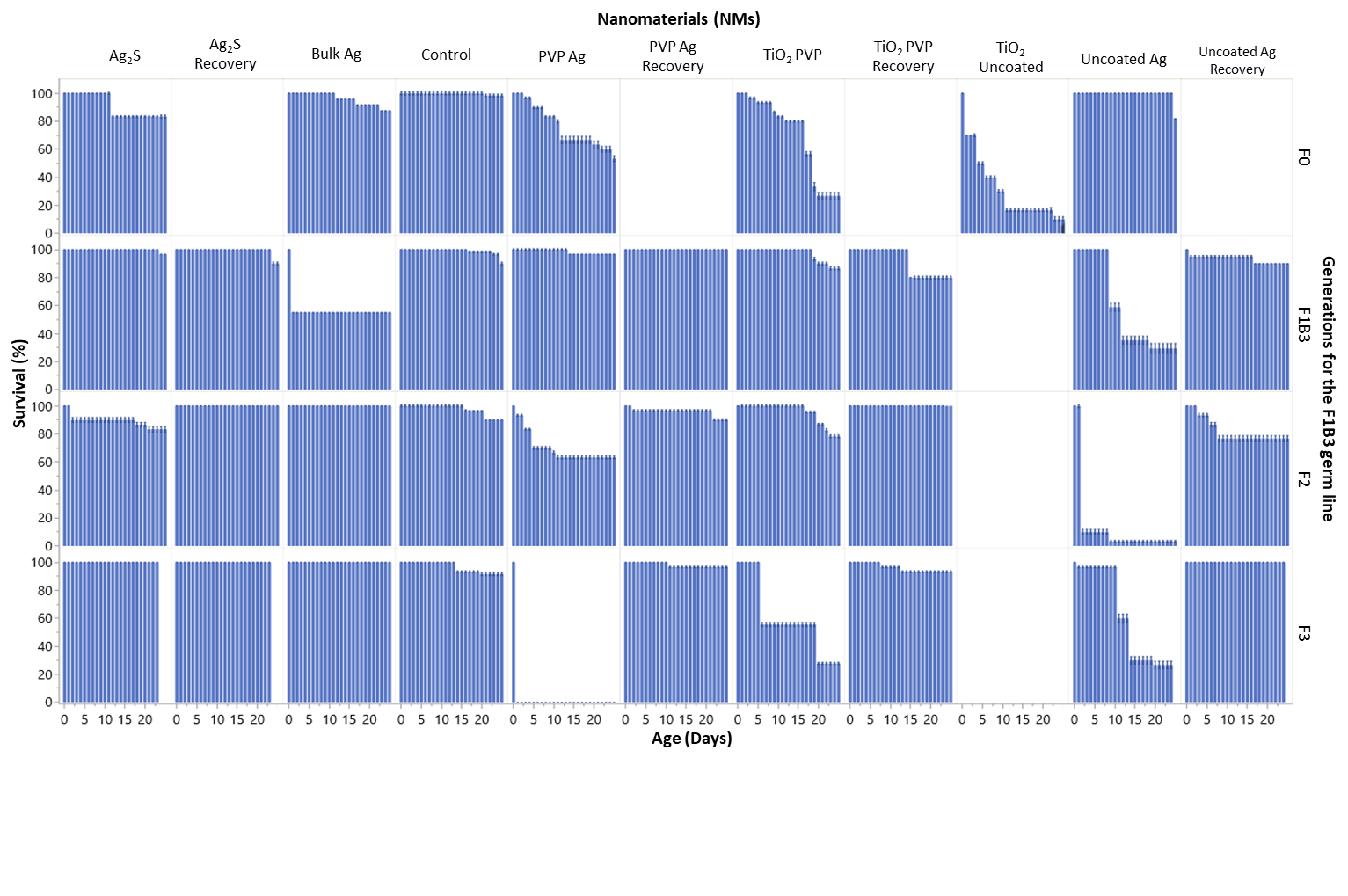
**

**Figure SI.4:** Multigenerational longevity for daphnids both continuously exposed to each of the Ag and TiO_2_ NMs, and those in the recovery groups, for the subsequent generations of the F1B3 germ lines. The Y-axis indicates the average survival (%) versus the *Daphnia* age (in days at the time of measurement) on the X-axis. F0 = Parent exposure to the particular NM is noted at the top of the plots, and the following plots are split for each generation. Data are mean ± SD.

**
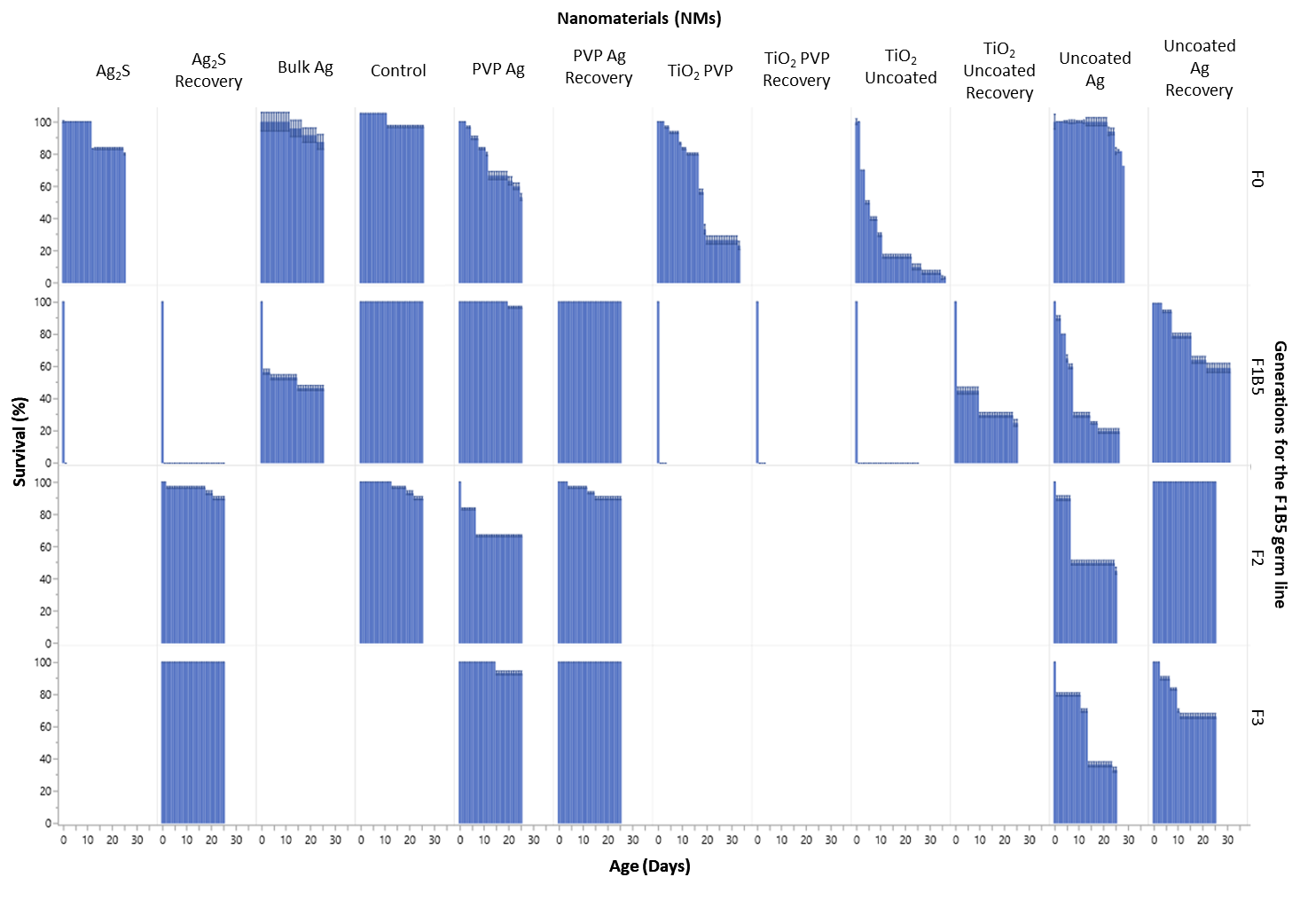
**

**Figure SI.5:** Multigenerational longevity for daphnids both continuously exposed to each of the Ag and TiO_2_ NMs, and those in the recovery groups, for the subsequent generations of the F1B5 germ lines. The Y-axis indicates the average survival (%) versus the *Daphnia* age (in days at the time of measurement) on the X-axis. F0 = Parent exposure to the particular NM is noted at the top of the plots, and the following plots are split for each generation. Data are mean ± SD.

**
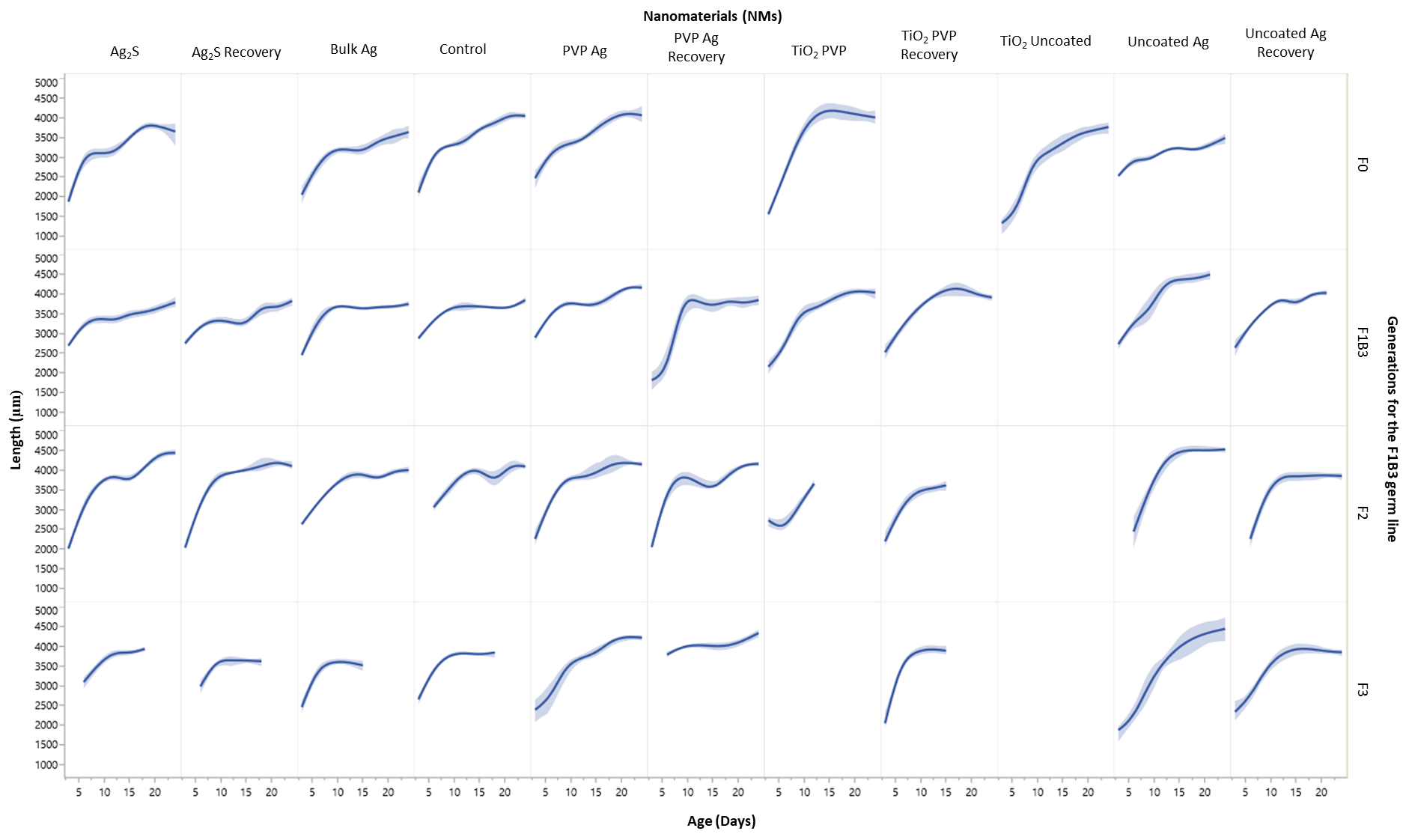
**

**Figure SI.6:** Multigenerational growth for daphnids both continuously exposed to each of the Ag and TiO_2_ NMs, and those in the recovery following F0 parent exposure, for the subsequent generations of the F1B3 germ lines. The Y-axis indicates the average daphnid length (µm) (length was determined measuring from the apex of the helmet to the base of the tail) versus time (daphnid age) on the X-axis. F0 = Parent exposure to the particular NM is noted at the top of the plots. Data are mean ± SD. Data for F1B1 and F1B5 germline are absent due to non-sampling.

**2.3 Reproduction**


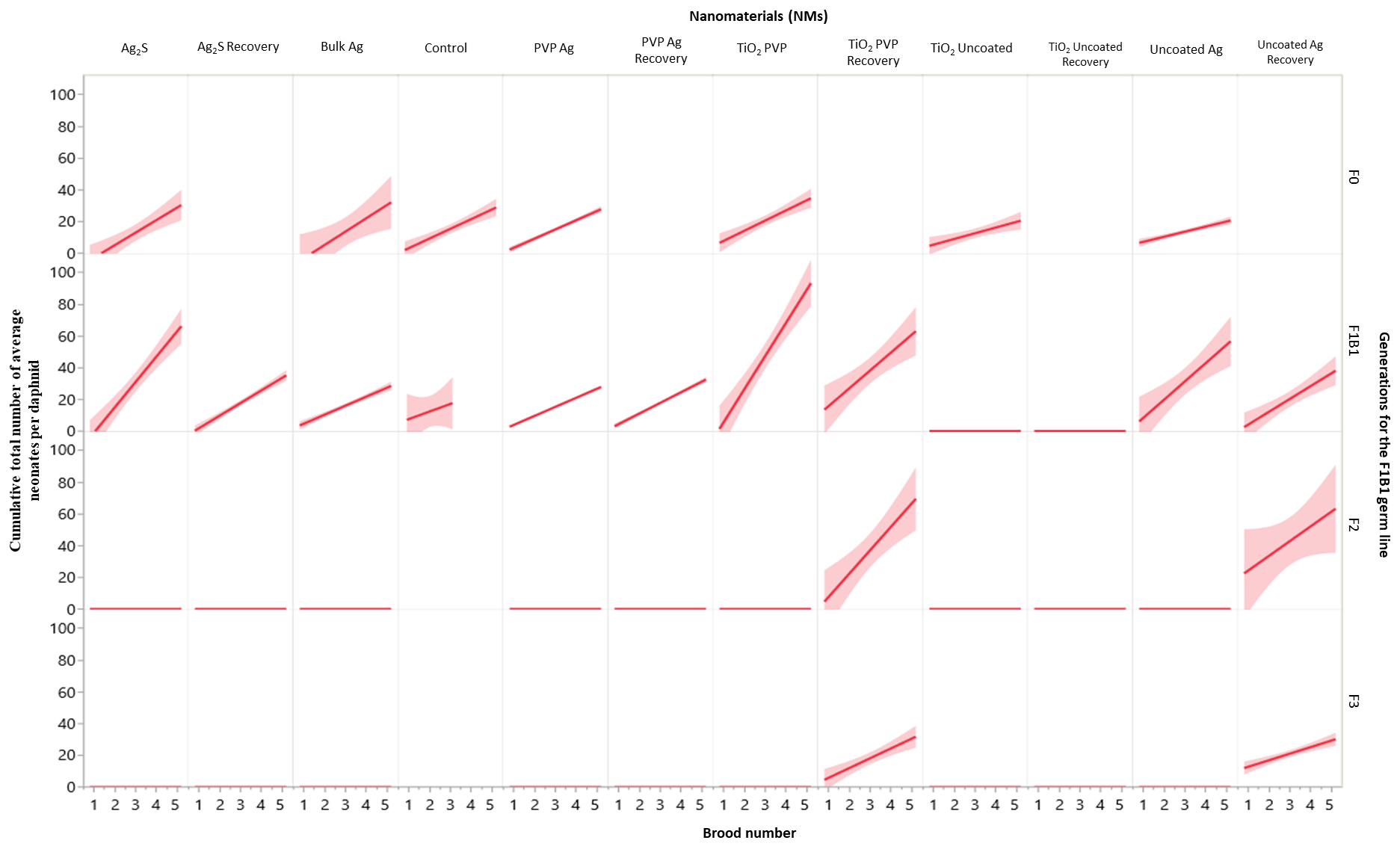


**Figure SI.7:** The average cumulative total neonates per daphnid for the subsequent generations of the F1B1 germ lines (continuous exposure and recovery). Data is presented for daphnids continuously exposed to each of the Ag and TiO_2_ NMs, and those in the recovery sets after removal from exposure. The Y-axis presents the average cumulative total neonates per daphnid versus time represented by brood number on the X-axis. F0 = Parent exposure to the particular NM is noted at the top of the plots. The graph splits horizontally by each of the generations and vertically by the NM exposure condition. The shaded areas around the lines are the 95% confidence bands.


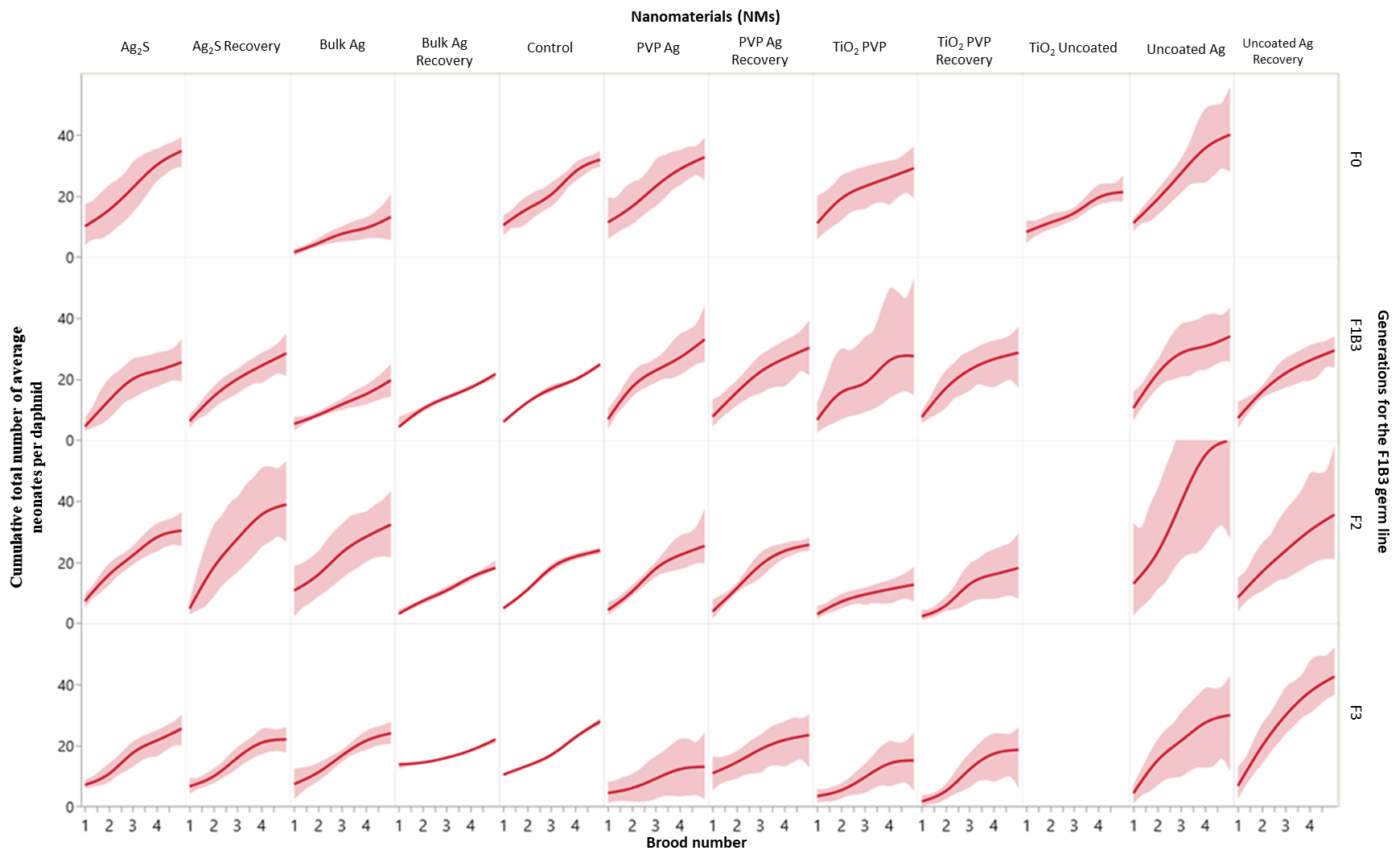


**Figure SI.8:** The average cumulative total neonates per daphnid for the subsequent generations of the F1B3 germ lines (continuous exposure and recovery). Data is presented for daphnids continuously exposed to each of the Ag and TiO_2_ NMs, and those in the recovery sets after removal from exposure. The Y-indicates the average cumulative total neonates per daphnid versus time represented as brood number on the X-axis. F0 = Parent exposure to the particular NM is noted at the top of the plots. The graph splits horizontally by each of the generations and vertically by the NM exposure condition. The shaded areas around the lines are the 95% confidence bands.


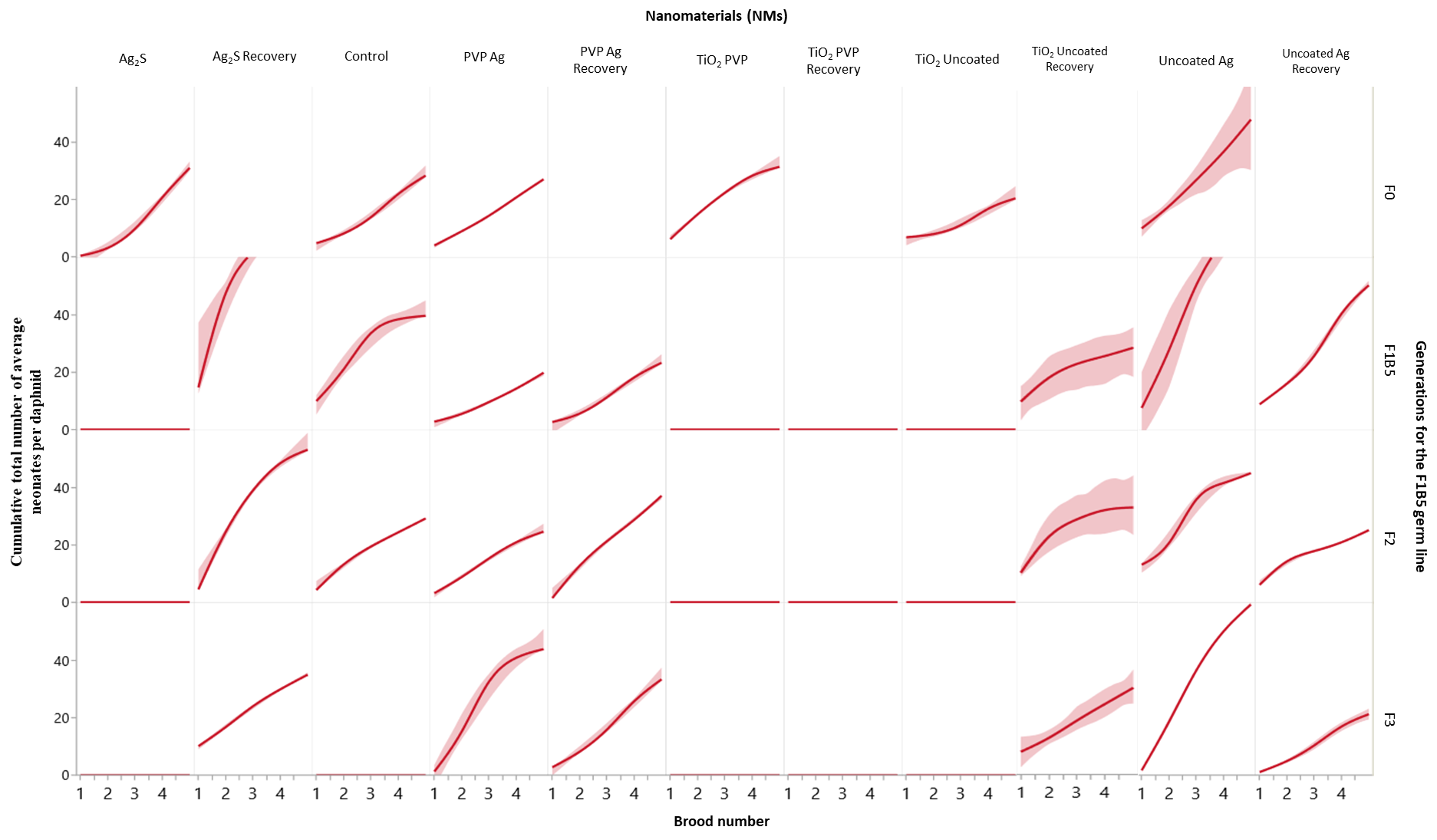


**Figure SI.9:** The average cumulative total neonates per daphnid for the subsequent generations of the F1B5 germ lines (continuous exposure and recovery). Data is presented for daphnids continuously exposed to each of the Ag and TiO_2_ NMs, and those in the recovery sets after removal from exposure. The Y-axis indicates the average cumulative total neonates per daphnid versus time indicated as brood number on the X-axis. F0 = Parent exposure to the particular NM is noted at the top of the plots. The graph is split horizontally by each of the generations and vertically by the NM exposure condition. The shaded areas around the lines are the 95% confidence bands.

| **Table SI.8: Reproduction data for F1B1 and subsequent generations** | | | | | | | | | | |
| --- | --- | --- | --- | --- | --- | --- | --- | --- | --- | --- |
| **NM and Generation** | **Time to 1^st^ brood (Days)** | **Av. Number offspring/adult in 1^st^ brood** | **Time to 2^nd^ brood (Days)** | **Av. Number offspring/adult in 2^nd^ brood** | **Time to 3^rd^ brood (Days)** | **Av. Number offspring/adult in 3^rd^ brood** | **Time to 4^th^ brood (Days)** | **Av. Number offspring/adult in 4^th^ brood** | **Time to 5^th^ brood (Days)** | **Av. Number offspring/adult in 5^th^ brood** |
| **Control F0** | 11 | 5 | 15 | 3 | 18 | 5 | 22 | 10 | 24 | 5 |
| **Control F1B1** | 12 | 7 | 15 | 6 | 19 | 3 | NA | NA | NA | NA |
| **Control F2** | NA | NA | NA | NA | NA | NA | NA | NA | NA | NA |
| **Control F3** | NA | NA | NA | NA | NA | NA | NA | NA | NA | NA |
| **Bulk Ag F0** | 11 | 0 | 15 | 4 | 17 | 8 | 21 | 2 | 23 | 8 |
| **Bulk Ag F1B1** | 10 | 4 | 13 | 8 | 15 | 5 | 18 | 5 | 21 | 6 |
| **PVP Ag F0** | 11 | 4 | 15 | 5 | 18 | 5 | 21 | 7 | 24 | 6 |
| **PVP Ag F1B1** | 10 | 4 | 13 | 6 | 18 | 6 | 21 | 5 | 25 | 6 |
| **PVP Ag Recovery F1B1** | 10 | 4 | 13 | 7 | 16 | 8 | 19 | 6 | 23 | 6 |
| **Ag_2_S F0** | 11 | 1 | 15 | 2 | 17 | 6 | 21 | 13 | 24 | 9 |
| **Ag_2_S F1B1** | 11 | 2 | 13 | 13 | 17 | 11 | 19 | 19 | 22 | 22 |
| **Ag_2_S Recovery F1B1** | 10 | 3 | 12 | 6 | 15 | 7 | 20 | 10 | 23 | 8 |
| **Uncoated Ag F0** | 13 | 7 | 18 | 3 | 22 | 5 | 25 | 1 | 28 | 4 |
| **Uncoated Ag F1B1** | 11 | 3 | 14 | 24 | 18 | 5 | 21 | 15 | 25 | 3 |
| **Uncoated Ag Recovery F1B1** | 10 | 1 | 13 | 16 | 17 | 2 | 20 | 10 | 24 | 7 |
| **Uncoated Ag Recovery F2** | 12 | 13 | 17 | 33 | 21 | 1 | 24 | 3 | 28 | 7 |
| **Uncoated Ag Recovery F3** | 10 | 14 | 14 | 2 | 17 | 4 | 20 | 7 | 24 | 2 |
| **TiO_2_ Uncoated F0** | 17 | 7 | 23 | 1 | 30 | 2 | 33 | 8 | 36 | 2 |
| **TiO_2_ Uncoated F1B1** | NA | NA | NA | NA | NA | NA | NA | NA | NA | NA |
| **TiO_2_ PVP F0** | 12 | 6 | 14 | 9 | 20 | 7 | 29 | 7 | 33 | 2 |
| **TiO_2_ PVP F1B1** | 10 | 2 | 15 | 2 | 18 | 7 | 21 | 7 | 31 | 11 |
| **TiO_2_ PVP Recovery F1B1** | 12 | 11 | 15 | 17 | 18 | 18 | 21 | 3 | 24 | 8 |
| **TiO_2_ PVP Recovery F2** | 11 | 5 | 14 | 13 | 17 | 30 | 21 | 4 | 26 | 10 |
| **TiO_2_ PVP Recovery F3** | 9 | 4 | 14 | 8 | 17 | 9 | 20 | 4 | 23 | 3 |

***NA: No data is presented where there is either mortality, no neonates, where daphnids failed to become gravid or no data collected**

| **Table SI.9: Reproduction data for F1B3 and subsequent generations** | | | | | | | | | | |
| --- | --- | --- | --- | --- | --- | --- | --- | --- | --- | --- |
| **NM and Generation** | **Time to 1^st^ brood (Days)** | **Av. Number offspring/adult in 1^st^ brood** | **Time to 2^nd^ brood (Days)** | **Av. Number offspring/adult in 2^nd^ brood** | **Time to 3^rd^ brood (Days)** | **Av. Number offspring/adult in 3^rd^ brood** | **Time to 4^th^ brood (Days)** | **Av. Number offspring/adult in 4^th^ brood** | **Time to 5^th^ brood (Days)** | **Av. Number offspring/adult in 5^th^ brood** |
| **Control F0** | 11 | 5 | 15 | 3 | 18 | 5 | 22 | 10 | 24 | 5 |
| **Control F1B3** | 11 | 6 | 15 | 6 | 19 | 3 | 22 | 4 | 25 | 5 |
| **Control F2** | 12 | 6 | 14 | 6 | 19 | 9 | 23 | 3 | 26 | 2 |
| **Control F3** | 12 | 11 | 15 | 3 | 21 | 2 | 23 | 6 | 25 | 4 |
| **Bulk Ag F0** | 11 | 0 | 15 | 4 | 17 | 8 | 21 | 2 | 23 | 8 |
| **Bulk Ag F1B3** | 12 | 7 | 18 | 0.5 | 21 | 6 | 25 | 5 | 28 | 7 |
| **Bulk Ag F2** | 12 | 20 | 15 | 4 | 19 | 11 | 23 | 4 | 26 | 5 |
| **Bulk Ag F3** | 11 | 2 | 14 | 5 | 17 | 9 | 20 | 2 | 23 | 2 |
| **PVP Ag F0** | 11 | 4 | 15 | 5 | 18 | 5 | 21 | 7 | 24 | 6 |
| **PVP Ag F1B3** | 10 | 12 | 13 | 4 | 19 | 13 | 26 | 13 | 30 | 7 |
| **PVP Ag F2** | 7 | 2 | 10 | 12 | 14 | 7 | NA | NA | NA | NA |
| **PVP Ag F3** | NA | NA | NA | NA | NA | NA | NA | NA | NA | NA |
| **PVP Ag Recovery F1B3** | 13 | 16 | 16 | 2 | 19 | 13 | 26 | 7 | 30 | 5 |
| **PVP Ag Recovery F2** | 15 | 1 | 21 | 9 | 24 | 6 | 28 | 7 | NA | NA |
| **PVP Ag Recovery F3** | 11 | 4 | 14 | 10 | 17 | 9 | 21 | 2 | 23 | 2 |
| **Ag_2_S F0** | 11 | 1 | 15 | 2 | 17 | 6 | 21 | 13 | 24 | 9 |
| **Ag_2_S F1B3** | 11 | 3 | 15 | 0 | 19 | 9 | 23 | 3 | 26 | 6 |
| **Ag_2_S F2** | 10 | 5 | 14 | 12 | 18 | 8 | 21 | 11 | 25 | 4 |
| **Ag_2_S F3** | 12 | 7.3 | 17 | 5 | 20 | 10 | 24 | 2 | 25 | 5 |
| **Ag_2_S Recovery F1B3** | 11 | 10 | 14 | 3 | 19 | 12 | 23 | 6 | 26 | 9 |
| **Ag_2_S Recovery F2** | 10 | 5 | 14 | 12 | 18 | 9 | 21 | 13 | 28 | 3 |
| **Ag_2_S Recovery F3** | 12 | 3 | 14 | 3 | 17 | 5 | 20 | 7 | 24 | 3 |
| **Uncoated Ag F0** | 13 | 7 | 18 | 3 | 22 | 5 | 25 | 1 | 28 | 4 |
| **Uncoated Ag F1B3** | 12 | 19 | 16 | 9 | 21 | 20 | 25 | 20 | 28 | 17 |
| **Uncoated Ag F2** | 9 | 3 | 12 | 11 | 15 | 29 | 18 | 32 | 20 | 6 |
| **Uncoated Ag F3** | 11 | 1 | 14 | 14 | 17 | 5 | 20 | 10 | NA | NA |
| **Uncoated Ag Recovery F1B3** | 12 | 16 | 16 | 6 | 20 | 11 | 22 | 1 | 26 | 8 |
| **Uncoated Ag Recovery F2** | 13 | 10 | 17 | 3 | 19 | 4 | 21 | 6 | 24 | 1 |
| **Uncoated Ag Recovery F3** | 12 | 5 | 15 | 12 | 17 | 14 | 21 | 5 | 24 | 5 |
| **TiO_2_ Uncoated F0** | 17 | 7 | 23 | 1 | 30 | 2 | 33 | 8 | 36 | 2 |
| **TiO_2_ Uncoated F1B3** | 12 | 7 | 19 | 5 | 23 | 3 | 30 | 1 | NA | NA |
| **TiO_2_ Uncoated Recovery F1B3** | 9 | 5 | 15 | 20 | NA | NA | NA | NA | NA | NA |
| **TiO_2_ PVP F0** | 12 | 6 | 14 | 9 | 20 | 7 | 29 | 7 | 33 | 2 |
| **TiO_2_ PVP F1B3** | 12 | 7 | 19 | 5 | 23 | 3 | 30 | 1 | NA | NA |
| **TiO_2_ PVP F2** | 16 | 7 | 20 | 0 | 22 | 5 | 24 | 4 | 27 | 6 |
| **TiO_2_ PVP F3** | 13 | 7 | 16 | 2 | 20 | 2 | 25 | 0.6 | 28 | 1 |
| **TiO_2_ PVP Recovery F1B3** | 11 | 11 | 15 | 10 | 18 | 14 | 22 | 1 | 25 | 6 |
| **TiO_2_ PVP Recovery** | 9 | 2 | 18 | 2 | 21 | 1 | 23 | 2 | NA | NA |
| **TiO_2_ PVP Recovery** | 8 | 0.3 | 13 | 7 | 16 | 15 | 20 | 2 | 23 | 2 |

***NA: No data is presented where there is either mortality, no neonates where daphnids failed to become gravid or no data collected**

| **Table SI.10: Reproduction data for F1B5 and subsequent generations** | | | | | | | | | | |
| --- | --- | --- | --- | --- | --- | --- | --- | --- | --- | --- |
| **NM and Generation** | **Time to 1^st^ brood (Days)** | **Av. Number offspring/adult in 1^st^ brood** | **Time to 2^nd^ brood (Days)** | **Av. Number offspring/adult in 2^nd^ brood** | **Time to 3^rd^ brood (Days)** | **Av. Number offspring/adult in 3^rd^ brood** | **Time to 4^th^ brood (Days)** | **Av. Number offspring/adult in 4^th^ brood** | **Time to 5^th^ brood (Days)** | **Av. Number offspring/adult in 5^th^ brood** |
| **Control F0** | 11 | 5 | 15 | 3 | 18 | 5 | 22 | 10 | 24 | 5 |
| **Control F1B5** | 11 | 10 | 15 | 8 | 18 | 18 | 22 | 1 | 25 | 2 |
| **Control F2** | 13 | 4 | 15 | 10 | 19 | 6 | 22 | 5 | 25 | 5 |
| **Control F3** | NA | NA | NA | NA | NA | NA | NA | NA | NA | NA |
| **Bulk Ag F0** | 11 | 0 | 15 | 4 | 17 | 8 | 21 | 2 | 23 | 8 |
| **Bulk Ag F1B5** | NA | NA | NA | NA | NA | NA | NA | NA | NA | NA |
| **PVP Ag F0** | 11 | 4 | 15 | 5 | 18 | 5 | 21 | 7 | 24 | 6 |
| **PVP Ag F1B5** | 11 | 3 | 15 | 2 | 20 | 5 | 23 | 4 | 26 | 6 |
| **PVP Ag F2** | 11 | 3 | 14 | 5 | 17 | 7 | 20 | 6 | 23 | 3 |
| **PVP Ag F3** | 8 | 2 | 12 | 10 | 15 | 24 | 19 | 4 | 22 | 3 |
| **PVP Ag Recovery F1B5** | 11 | 3 | 13 | 2 | 18 | 6 | 21 | 8 | 25 | 4 |
| **PVP Ag Recovery F2** | 10 | 1 | 14 | 12 | 17 | 8 | 20 | 7 | 23 | 9 |
| **PVP Ag Recovery F3** | 11 | 3 | 13 | 5 | 17 | 7 | 21 | 12 | 24 | 6 |
| **Ag_2_S F0** | 11 | 1 | 15 | 2 | 17 | 6 | 21 | 13 | 24 | 9 |
| **Ag_2_S F1B5** | NA | NA | NA | NA | NA | NA | NA | NA | NA | NA |
| **Ag_2_S Recovery F1B5** | 13 | 12 | 16 | 41 | 19 | 7 | 22 | 9 | 26 | 2 |
| **Ag_2_S Recovery F2** | 11 | 4 | 14 | 22 | 18 | 13 | 21 | 11 | 25 | 4 |
| **Ag_2_S Recovery F3** | 13 | 10 | 17 | 6 | 19 | 8 | 23 | 5 | 26 | 5 |
| **Uncoated Ag F0** | 13 | 7 | 18 | 3 | 22 | 5 | 25 | 1 | 28 | 4 |
| **Uncoated Ag F1B5** | 12 | 8 | 15 | 36 | 18 | 13 | 22 | 16 | 26 | 11 |
| **Uncoated Ag F2** | 11 | 14 | 14 | 5 | 18 | 20 | 22 | 3 | 25 | 4 |
| **Uncoated Ag F3** | 10 | 2 | 14 | 16 | 18 | 19 | 21 | 13 | 24 | 9 |
| **Uncoated Ag Recovery F1B5** | 12 | 9 | 15 | 7 | 18 | 8 | 22 | 17 | 26 | 8 |
| **Uncoated Ag Recovery F2** | 15 | 6 | 18 | 9 | 22 | 3 | 25 | 2 | 28 | 4 |
| **Uncoated Ag Recovery F3** | 10 | 2 | 12 | 4 | 14 | 4 | 18 | 7 | 20 | 8 |
| **TiO_2_ Uncoated F0** | 17 | 7 | 23 | 1 | 30 | 2 | 33 | 8 | 36 | 2 |
| **TiO_2_ Uncoated F1B5** | NA | NA | NA | NA | NA | NA | NA | NA | NA | NA |
| **TiO_2_ Uncoated Recovery F1B5** | 10 | 21 | 14 | 8 | 20 | 11 | 24 | 7 | 26 | 1 |
| **TiO_2_ Uncoated Recovery F2** | 12 | 0.6 | 15 | 2 | 19 | 4 | 22 | 9 | 27 | 9 |
| **TiO_2_ Uncoated Recovery F3** | 11 | 9 | 17 | 12 | 21 | 14 | 25 | 22 | 28 | 22 |
| **TiO_2_ PVP F0** | 12 | 6 | 14 | 9 | 20 | 7 | 29 | 7 | 33 | 2 |
| **TiO_2_ PVP F1B5** | NA | NA | NA | NA | NA | NA | NA | NA | NA | NA |

***NA: No data is presented where there is either mortality, no neonates where daphnids failed to become gravid or no data collected**

**Gene expression**

**
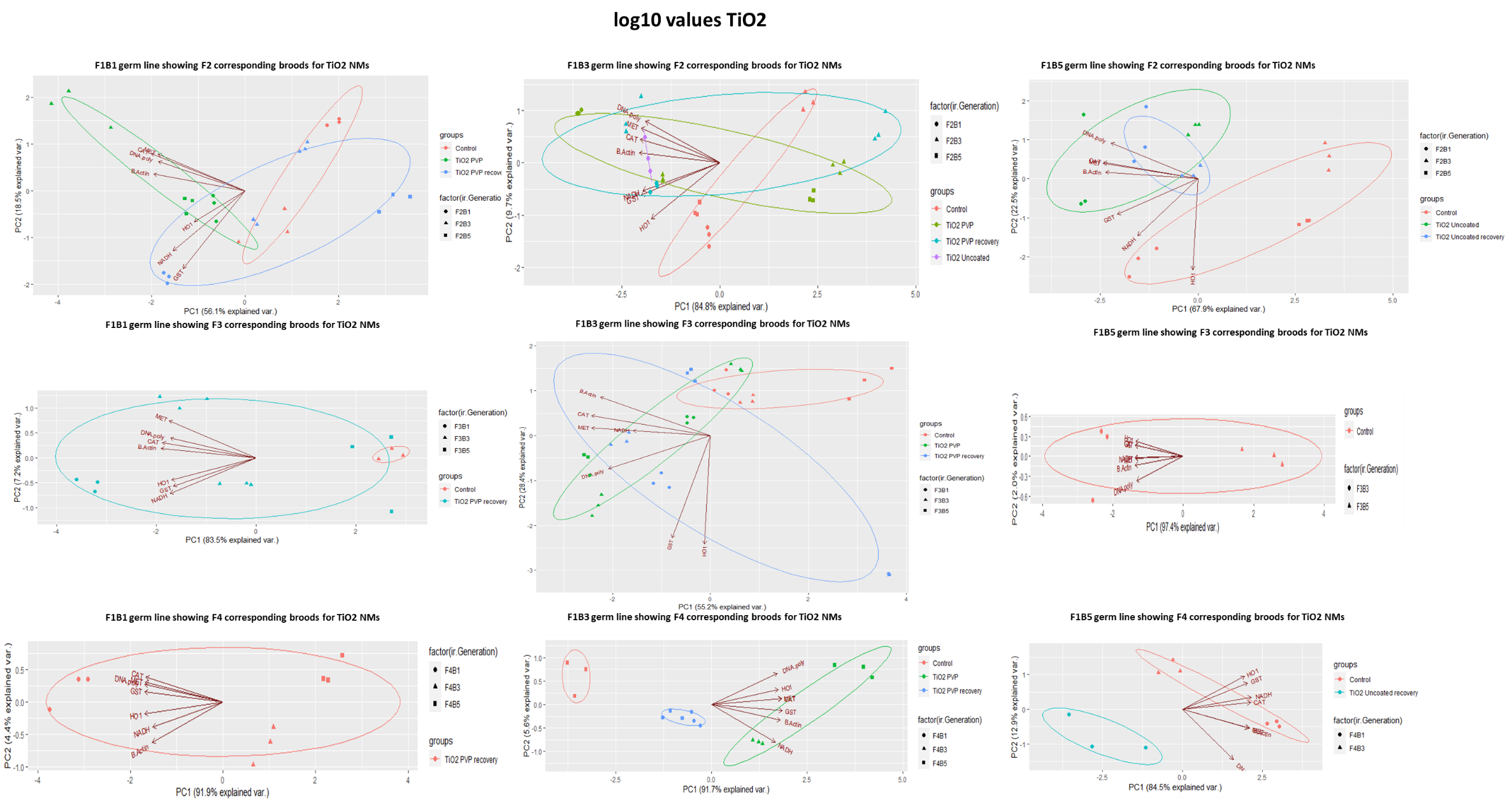
**

**Figure SI.10:** Multi-generational PCA plots for gene expression corresponding to the F2-F4 (inclusive of all broods 1,3 and 5 per generation) after exposure to TiO_2_ NMs.

**
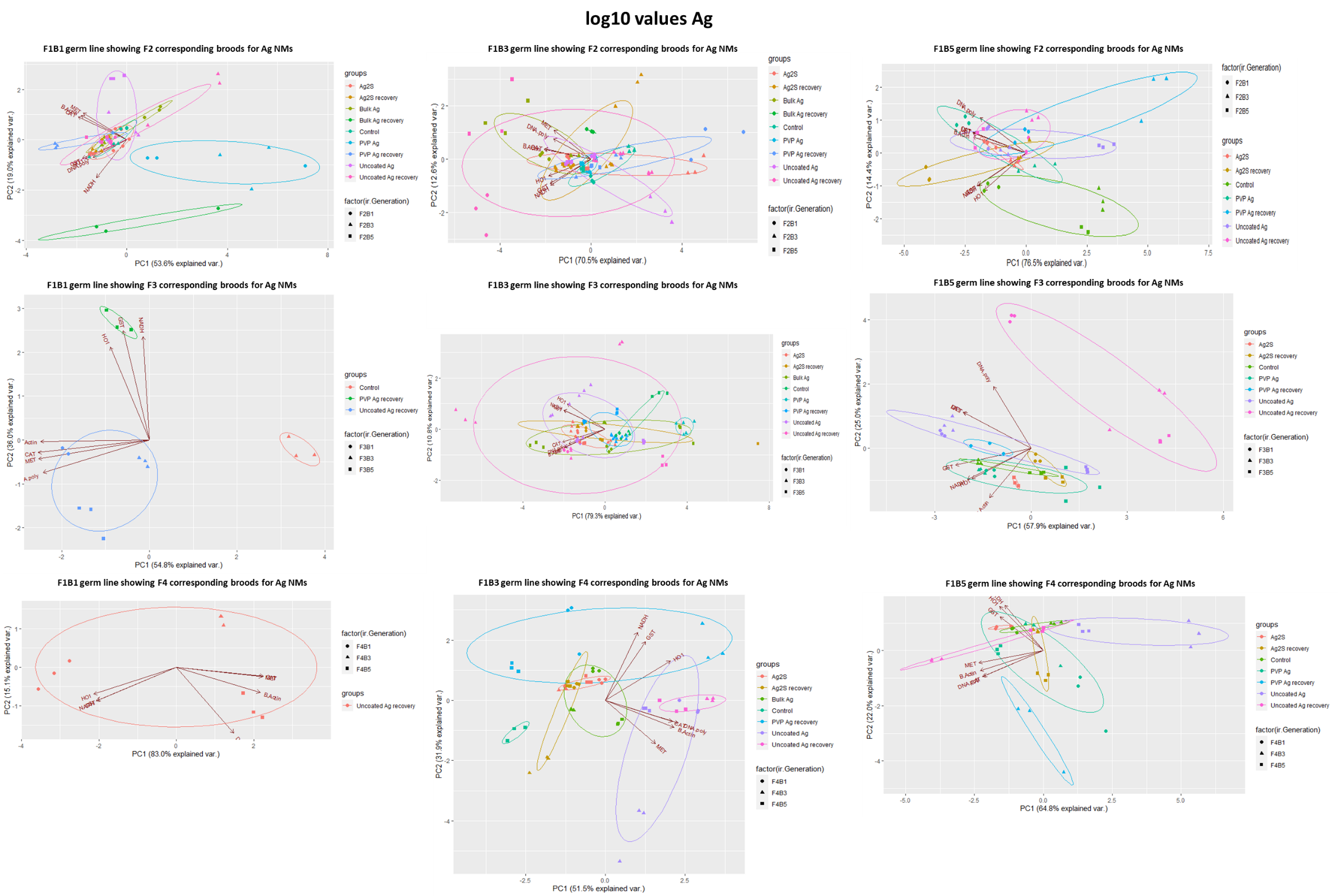
**

**Figure SI.11:** Multi-generational PCA plots for gene expression corresponding to the F2-F4 generations (inclusive of all broods 1, 3 and 5 per generation) after exposure to Ag NMs.


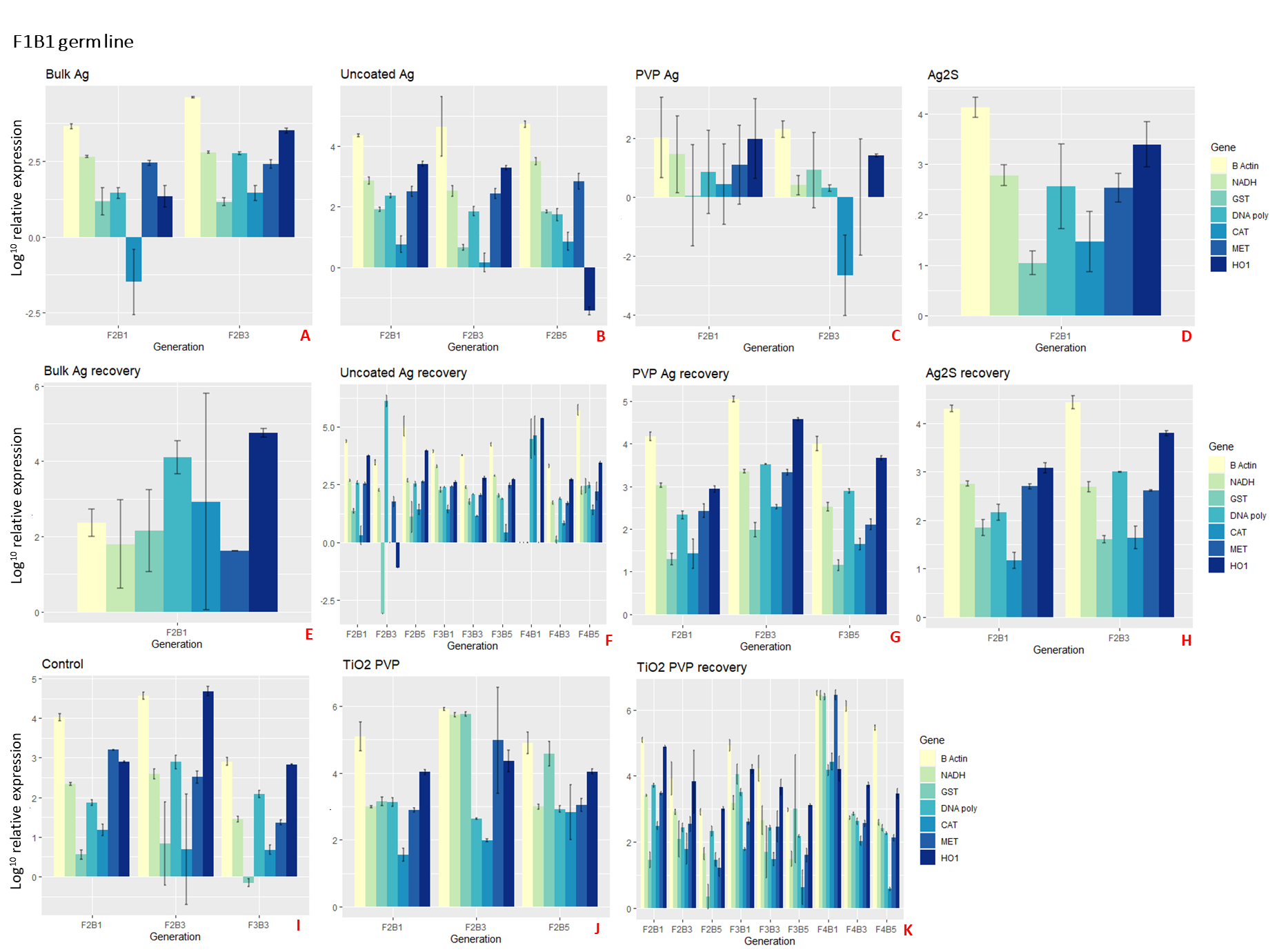


**Figure SI.12:** F1B1 Germline gene expression over generations F2 and F3 for the continuously exposed and recovery daphnids for each of the Ag and TiO_2_ NMs tested.

**
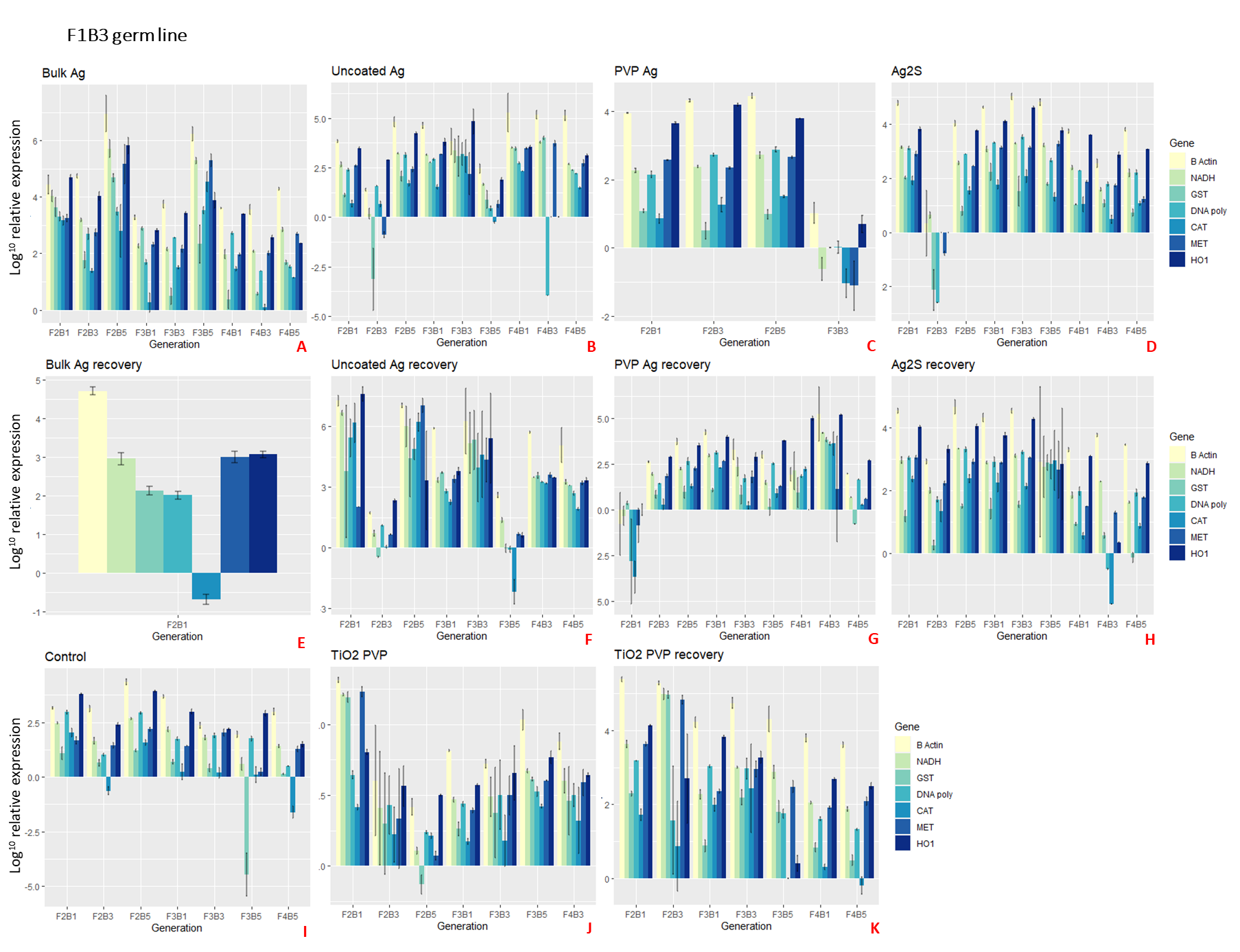
**

**Figure SI.13:** F1B3 Germline gene expression over generations F2 and F3 for the continuously exposed and recovery daphnids for each of the Ag and TiO_2_ NMs tested.

**
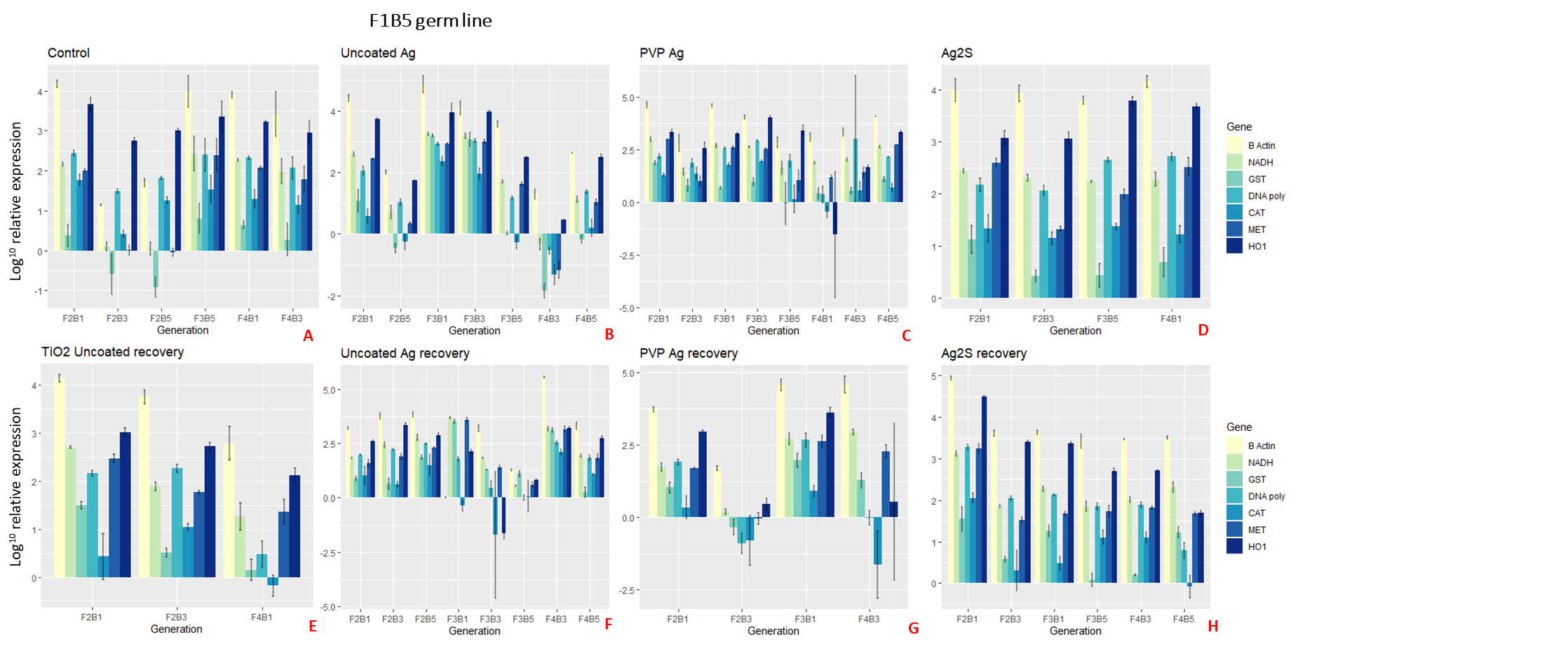
**

**Figure SI.14:** F1B5 Germline gene expression over generations F2 and F3 for the continuously exposed and recovery daphnids for each of the Ag NMs tested.


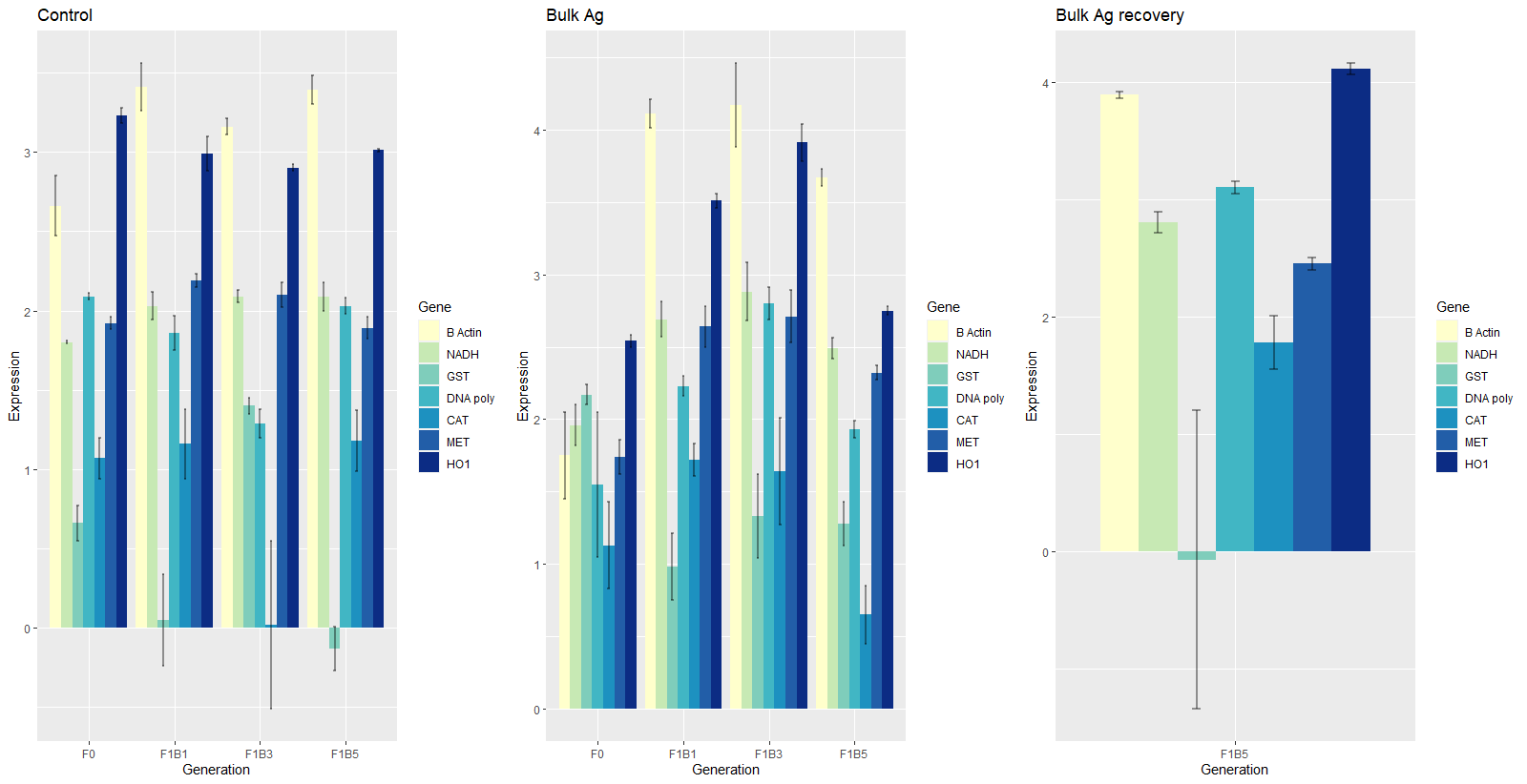


**Figure SI.15:** Relative gene expression for the Bulk Ag NM exposure in the three broods of the F1B1 and F1B3 generations. The most responsive genes that were up regulated were β-actin and HO1.

**Table SI.11 PCA loading for the F0 generation exposed to the Ag NMs**

PC1 PC2 PC3 PC4 PC5 PC6 PC7

| B.Actin -0.21083820 0.56487659 -0.4785253 -0.10818440 0.50112126 0.37751202 -0.04618998  NADH 0.52530331 0.10627533 -0.2118964 -0.21946707 0.22932571 -0.56276986 -0.50039576  GST 0.54892090 0.06980814 0.1354449 -0.08116179 0.34305391 -0.01401616 0.74229218  DNA.poly -0.28111191 -0.32689253 0.4089929 -0.65875402 0.44368090 0.05912855 -0.11196590  CAT -0.06615626 0.63581525 0.2480912 -0.49974960 -0.49568145 -0.14494462 0.11556111  MET -0.18356848 0.38183760 0.5987483 0.49941728 0.35795169 -0.26226629 -0.12518965  HO1 0.51106906 0.08847093 0.3470506 0.02046862 -0.08898859 0.66879710 -0.39358571  Importance of components:  PC1 PC2 PC3 PC4 PC5 PC6 PC7  Standard deviation 1.7483 1.2874 1.0922 0.8966 0.40405 0.28551 0.21085  Proportion of Variance 0.4367 0.2368 0.1704 0.1148 0.02332 0.01164 0.00635  Cumulative Proportion 0.4367 0.6734 0.8438 0.9587 0.98200 0.99365 1.00000 |
| --- |

**Table SI.12 PCA loading for the F0 generation exposed to the TiO_2_ NMs**

PC1 PC2 PC3 PC4 PC5 PC6 PC7

B.Actin 0.3804615 0.31466822 -0.1323604 0.7224491 -0.3375481 0.03239862 0.3190495

NADH 0.3666515 -0.75041638 0.0297501 -0.1668700 -0.4769894 -0.08562039 0.1971333

GST 0.3807347 -0.05912155 -0.5231852 -0.2067530 0.4536149 0.49823536 0.2847302

DNA.poly 0.3769540 -0.07354398 0.7765402 0.1229962 0.4569201 0.07210148 0.1427575

CAT 0.3760415 0.48914864 0.2101661 -0.4961243 -0.4366346 0.30484523 -0.2131594

MET 0.3819043 0.23845204 -0.1804681 -0.2637518 0.1915238 -0.80313657 0.1159578

HO1 0.3827589 -0.18118867 -0.1676895 0.2768439 0.1291077 -0.01494707 -0.8360692

Importance of components:

PC1 PC2 PC3 PC4 PC5 PC6 PC7

Standard deviation 2.6026 0.39606 0.24363 0.06877 0.06217 0.03436 0.02453

Proportion of Variance 0.9676 0.02241 0.00848 0.00068 0.00055 0.00017 0.00009

Cumulative Proportion 0.9676 0.99004 0.99852 0.99919 0.99975 0.99991 1.00000

**Table SI.13 PCA loading for the three broods of the F1 generation exposed to TiO_2_ NMs**

PC1 PC2 PC3 PC4 PC5 PC6 PC7

B.Actin 0.3199804 0.42827567 0.72352583 0.395587893 -0.1582487 0.07133217 0.06389154

NADH 0.1988438 -0.77792410 0.10168348 0.342255015 -0.1060131 0.39798829 0.24121311

GST 0.3770921 -0.40948834 0.35784341 -0.296127397 0.1534820 -0.58546511 -0.32871172

DNA.poly 0.4096178 0.05738264 -0.44096331 0.194324230 -0.7161218 -0.25830434 -0.13099030

CAT 0.4451319 0.12789426 -0.23023958 -0.003333084 0.3645788 -0.25642201 0.73062260

MET 0.4270649 0.11850900 -0.29589782 0.305636891 0.5118534 0.28705757 -0.52745219

HO1 0.4093430 0.10019607 0.05571808 -0.712385684 -0.1800552 0.52808354 0.02256222

Importance of components:

PC1 PC2 PC3 PC4 PC5 PC6 PC7

Standard deviation 2.1057 1.1087 0.69759 0.61053 0.50610 0.40466 0.23980

Proportion of Variance 0.6334 0.1756 0.06952 0.05325 0.03659 0.02339 0.00822

Cumulative Proportion 0.6334 0.8090 0.87855 0.93180 0.96839 0.99178 1.00000

**Table SI.14 PCA loading for the three broods of the F1 generation exposed to Ag NMs**

| PC1 PC2 PC3 PC4 PC5 PC6 PC7  B.Actin 0.3848492 0.2750222 -0.1336994 -0.66945936 0.54797139 -0.0312970 0.09460663  NADH 0.3896541 -0.3133274 0.4862035 0.07599493 0.21831891 0.2398218 -0.63454622  GST 0.3957909 -0.2724672 0.4705220 0.15124306 0.02742324 -0.1544847 0.70726817  DNA.poly 0.3747858 -0.2121655 -0.6313068 0.52840129 0.33119058 -0.1613032 -0.03254693  CAT 0.4017111 0.2665440 -0.1748362 0.07555822 -0.34997959 0.7640041 0.15848587  MET 0.3000274 0.7320498 0.2212143 0.29945095 -0.15076654 -0.4231076 -0.18365564  HO1 0.3893042 -0.3162175 -0.2151463 -0.38546938 -0.62960997 -0.3590257 -0.16812482  Importance of components:  PC1 PC2 PC3 PC4 PC5 PC6 PC7  Standard deviation 2.2861 0.9309 0.58879 0.50672 0.3579 0.33561 0.25129  Proportion of Variance 0.7466 0.1238 0.04952 0.03668 0.0183 0.01609 0.00902  Cumulative Proportion 0.7466 0.8704 0.91990 0.95658 0.9749 0.99098 1.00000 |
| --- |

**Table SI.15 PCA loading for the F1B1 germ line showing F2 corresponding broods when exposed to TiO_2_ NMs**

| PC1 PC2 PC3 PC4 PC5 PC6 PC7  B.Actin -0.4365460 0.1361330 -0.29817179 0.5263956971 -0.23311751 0.00386308 -0.6087182  NADH -0.3431032 -0.4997795 -0.22371036 -0.5764644617 0.32421042 0.04178069 -0.3785301  GST -0.2956719 -0.6466278 -0.19286614 0.3783993598 -0.13038169 -0.08399681 0.5385280  DNA.poly -0.4135345 0.2421598 0.27903257 0.1973848716 0.67712361 -0.42422766 0.1227291  CAT -0.4481777 0.3098372 -0.04408676 -0.0860196363 0.08373556 0.76826754 0.3107217  MET -0.4167221 0.3040850 -0.09479267 -0.4483858150 -0.51820229 -0.45432756 0.2211162  HO1 -0.2423935 -0.2577842 0.85735724 -0.0004632008 -0.29917020 0.12069103 -0.1888436  Importance of components:  PC1 PC2 PC3 PC4 PC5 PC6 PC7  Standard deviation 1.982 1.1388 0.9406 0.61440 0.56320 0.35135 0.27013  Proportion of Variance 0.561 0.1853 0.1264 0.05393 0.04531 0.01764 0.01042  Cumulative Proportion 0.561 0.7463 0.8727 0.92663 0.97194 0.98958 1.00000 |
| --- |
| **Table SI.16 PCA loading for F1B1 germ line showing F3 corresponding broods for TiO_2_ NMs** |

PC1 PC2 PC3 PC4 PC5 PC6 PC7

B.Actin -0.4086492 0.1405292 -0.06637372 0.05232325 0.16576323 -0.49488875 -0.73056273

NADH -0.3698773 -0.5197416 -0.05068007 -0.62825897 0.41770221 0.02611599 0.14361207

GST -0.3564105 -0.4133475 0.55694594 0.59757381 0.11334524 0.14240481 0.04130221

DNA.poly -0.3680495 0.2900801 0.52947180 -0.43090705 -0.55154745 0.10029692 -0.01038067

CAT -0.4027524 0.2246215 -0.18002237 0.17250172 -0.03147266 -0.54123132 0.65669543

MET -0.3732390 0.5457898 -0.16693360 0.08815157 0.41476788 0.59536345 0.02604866

HO1 -0.3635806 -0.3267663 -0.58499745 0.14916453 -0.55495294 0.27690343 -0.10914498

Importance of components:

PC1 PC2 PC3 PC4 PC5 PC6 PC7

Standard deviation 2.4179 0.70836 0.64068 0.34559 0.30537 0.1472 0.08424

Proportion of Variance 0.8352 0.07168 0.05864 0.01706 0.01332 0.0031 0.00101

Cumulative Proportion 0.8352 0.90687 0.96551 0.98257 0.99589 0.9990 1.00000

**Table SI.17 PCA loading for F1B1 germ line showing F4 corresponding broods for TiO_2_ NMs**

| PC1 PC2 PC3 PC4 PC5 PC6 PC7  B.Actin -0.3530312 -0.6595680 -0.51204101 -0.41174768 0.05123503 -0.010709407 0.07666279  NADH -0.3494969 -0.4048356 0.83598301 -0.07909026 0.08143932 0.043345841 0.01805451  GST -0.3903314 0.1579145 -0.14291280 0.36506854 0.76522628 0.273094731 0.09409190  DNA.poly -0.3889568 0.2868518 -0.02599975 -0.25431140 -0.03901085 0.002032883 -0.83639250  CAT -0.3841122 0.3977302 0.03124969 -0.25084608 0.04692930 -0.691961849 0.38646424  MET -0.3875084 0.3156597 -0.01924198 -0.15575266 -0.45815813 0.621738915 0.35930332  HO1 -0.3897049 -0.1898075 -0.12845059 0.73421836 -0.43765086 -0.240937966 -0.08329265  Importance of components:  PC1 PC2 PC3 PC4 PC5 PC6 PC7  Standard deviation 2.5368 0.55704 0.48288 0.10357 0.09711 0.03271 0.006319  Proportion of Variance 0.9193 0.04433 0.03331 0.00153 0.00135 0.00015 0.000010  Cumulative Proportion 0.9193 0.96365 0.99696 0.99849 0.99984 0.99999 1.000000 |
| --- |

**Table SI.18 PCA loading for F1B3 germ line showing F2corresponding broods for TiO_2_ NMs**

| PC1 PC2 PC3 PC4 PC5 PC6 PC7  B.Actin -0.3970165 0.1106762 -0.3321851 0.3583781 0.59941906 -0.386446626 -0.28758070  NADH -0.3794827 -0.3005712 -0.5989456 -0.3312907 0.04525606 0.208201487 0.50176137  GST -0.3866472 -0.3476796 -0.1581715 0.1727597 -0.65317385 -0.050547533 -0.49554773  DNA.poly -0.3654580 0.4506684 0.1474069 -0.7055114 -0.10721574 -0.331971388 -0.14887282  CAT -0.3910719 0.2515096 0.2729337 0.4502021 -0.28904012 -0.241180069 0.60408520  MET -0.3863210 0.3768354 0.1259080 0.1142589 0.10344675 0.797725116 -0.18104316  HO1 -0.3363540 -0.6067641 0.6275544 -0.1359497 0.32599381 0.004231444 0.01032681  Importance of components:  PC1 PC2 PC3 PC4 PC5 PC6 PC7  Standard deviation 2.4361 0.82351 0.42736 0.35482 0.20940 0.1401 0.12332  Proportion of Variance 0.8478 0.09688 0.02609 0.01799 0.00626 0.0028 0.00217  Cumulative Proportion 0.8478 0.94468 0.97078 0.98876 0.99502 0.9978 1.00000 |
| --- |

**Table SI.19 PCA loading for F1B3 germ line showing F3 corresponding broods for TiO_2_ NMs**

PC1 PC2 PC3 PC4 PC5 PC6 PC7

B.Actin -0.45537992 0.24326765 -0.035633892 0.591820632 -0.34068829 0.265432987 -0.44204679

NADH -0.32059667 0.03118855 -0.852378097 -0.055018805 0.40748376 -0.007664398 0.02382871

GST -0.15929759 -0.64251059 -0.166554818 -0.407175651 -0.46419321 0.362757872 -0.14561349

DNA.poly -0.42258490 -0.20728729 0.463182918 -0.117750039 0.65389150 0.280526682 -0.20923955

CAT -0.49249128 0.12663147 0.125418384 0.005943271 -0.17846789 0.171122314 0.81517979

MET -0.49267049 0.04833660 0.118989037 -0.315189565 -0.18715428 -0.751418683 -0.20440096

HO1 -0.02492652 -0.68241358 -0.003836773 0.606369857 0.08922741 -0.353956509 0.18095445

Importance of components:

PC1 PC2 PC3 PC4 PC5 PC6 PC7

Standard deviation 1.9659 1.4111 0.8955 0.41517 0.32663 0.20726 0.14223

Proportion of Variance 0.5521 0.2845 0.1145 0.02462 0.01524 0.00614 0.00289

Cumulative Proportion 0.5521 0.8366 0.9511 0.97573 0.99097 0.99711 1.00000

**Table SI.20 PCA loading for F1B3 germ line showing F4 corresponding broods for TiO_2_ NMs**

PC1 PC2 PC3 PC4 PC5 PC6 PC7

B.Actin 0.3817494 -0.2872223 0.21511464 -0.81403335 0.2280941 -0.036010456 -0.09758313

NADH 0.3542109 -0.6902605 -0.13002146 0.36764183 -0.2199399 -0.276396590 -0.34819581

GST 0.3925785 -0.1109243 0.10232560 0.06051957 -0.3151059 -0.005813234 0.84859838

DNA.poly 0.3651268 0.5718066 0.32734903 0.02874946 -0.2872650 -0.537286863 -0.24604340

CAT 0.3900988 0.1065630 0.31892891 0.18718865 -0.1418990 0.780375327 -0.26568973

MET 0.3887141 0.1124232 -0.01402463 0.36078543 0.8259220 -0.100518326 0.11682643

HO1 0.3716058 0.2788943 -0.84692376 -0.18026182 -0.1245153 0.120447582 -0.06588780

Importance of components:

PC1 PC2 PC3 PC4 PC5 PC6 PC7

Standard deviation 2.5332 0.62351 0.33754 0.19757 0.17213 0.09307 0.05155

Proportion of Variance 0.9168 0.05554 0.01628 0.00558 0.00423 0.00124 0.00038

Cumulative Proportion 0.9168 0.97230 0.98857 0.99415 0.99838 0.99962 1.00000

**Table SI.21 PCA loading for F1B5 germ line showing F2 corresponding broods for TiO_2_ NMs**

| PC1 PC2 PC3 PC4 PC5 PC6 PC7  B.Actin -0.43960484 0.05553249 0.06303038 -0.50321451 0.53318837 -0.50939298 0.05191360  NADH -0.28739492 -0.46867857 -0.82958839 0.08097859 -0.03927651 -0.02384379 0.02930790  GST -0.38396381 -0.29434217 0.37031102 0.74333073 0.15732834 -0.22630396 -0.01726158  DNA.poly -0.41334786 0.29787449 0.01980842 -0.04998385 -0.77490798 -0.36237464 0.07566917  CAT -0.44994332 0.13840116 0.02917332 -0.08247470 0.02195619 0.43369458 -0.76300059  MET -0.45032754 0.12590311 0.08248090 -0.04265821 0.07571089 0.59905816 0.63884887  HO1 -0.02496408 -0.75291669 0.40327839 -0.42018670 -0.28760864 0.10227290 -0.01115591  Importance of components:  PC1 PC2 PC3 PC4 PC5 PC6 PC7  Standard deviation 2.180 1.2564 0.61383 0.44254 0.27415 0.12721 0.07009  Proportion of Variance 0.679 0.2255 0.05383 0.02798 0.01074 0.00231 0.00070  Cumulative Proportion 0.679 0.9044 0.95827 0.98625 0.99699 0.99930 1.00000 |
| --- |

**Table SI.22 PCA loading for F1B5 germ line showing F3 corresponding broods for TiO_2_ NMs (only controls samples available)**

| PC1 PC2 PC3 PC4 PC5 PC6  B.Actin -0.3804142 -0.26970104 0.30800143 0.1054041 0.35209179 0.02112173  NADH -0.3823973 -0.04458306 0.26984313 0.1693841 0.42099468 -0.53853470  GST -0.3797221 0.32074304 0.08317896 -0.5101297 0.32147108 0.58204232  DNA.poly -0.3690734 -0.71701874 -0.04342712 0.1208970 -0.34914674 0.35416405  CAT -0.3787116 0.32794939 0.44208840 -0.1608565 -0.68609899 -0.21853154  MET -0.3792740 -0.07353664 -0.70547607 -0.4148853 -0.03571379 -0.39308736  HO1 -0.3760080 0.44195394 -0.36107688 0.6981017 -0.03924875 0.20749724  Importance of components:  PC1 PC2 PC3 PC4 PC5 PC6  Standard deviation 2.6108 0.37202 0.18498 0.09914 0.03419 5.862e-16  Proportion of Variance 0.9738 0.01977 0.00489 0.00140 0.00017 0.000e+00  Cumulative Proportion 0.9738 0.99354 0.99843 0.99983 1.00000 1.000e+00 |
| --- |

**Table SI.23 PCA loading for F1B5 germ line showing F4 corresponding broods for TiO2 NMs**

PC1 PC2 PC3 PC4 PC5 PC6 PC7

B.Actin 0.3890142 -0.26144622 -0.588765466 0.04813299 -0.56825004 0.0240282463 -0.328430769

NADH 0.4047865 0.16677753 0.008949722 0.10817130 -0.04257590 -0.8153329032 0.360515658

GST 0.3818681 0.36653529 0.290783235 0.34993354 0.21582844 0.0009876168 -0.682815771

DNA.poly 0.2991088 -0.69573963 0.561931495 -0.29754360 -0.01430946 -0.0812140287 -0.124018584

CAT 0.4010446 0.09529063 -0.376649733 -0.58632826 0.57735938 0.1052873523 -0.002799135

MET 0.3939117 -0.26069691 -0.057423537 0.59082230 0.26083750 0.4016866533 0.441716945

HO1 0.3654084 0.45649336 0.328315017 -0.28660895 -0.47656317 0.3944877849 0.292270437

Importance of components:

PC1 PC2 PC3 PC4 PC5 PC6 PC7

Standard deviation 2.4325 0.9491 0.30922 0.21999 0.17191 0.08299 0.03955

Proportion of Variance 0.8453 0.1287 0.01366 0.00691 0.00422 0.00098 0.00022

Cumulative Proportion 0.8453 0.9740 0.98766 0.99457 0.99879 0.99978 1.00000

**Table SI.24 PCA loading for F1B1 germ line showing F2 corresponding broods for Ag NMs**

PC1 PC2 PC3 PC4 PC5 PC6 PC7

B.Actin -0.4147175 0.3682816 -0.089564268 0.394387884 0.12024236 0.48417572 0.52908422

NADH -0.2765794 -0.5777147 -0.272836675 0.565575459 -0.31145496 0.09533682 -0.29889922

GST -0.4024953 -0.2870663 -0.160541038 -0.049157853 0.76162642 -0.37800437 0.06662203

DNA.poly -0.3079083 -0.2701244 0.907108480 0.006516737 -0.05446340 0.04835091 0.06350542

CAT -0.4419275 0.3503496 -0.004137873 -0.238449529 0.09945153 0.31071568 -0.72017018

MET -0.3958021 0.4082911 -0.002748396 0.139958856 -0.39955628 -0.70433879 0.03612078

HO1 -0.3778147 -0.2929056 -0.262477608 -0.667590424 -0.36872848 0.13646349 0.31985608

Importance of components:

PC1 PC2 PC3 PC4 PC5 PC6 PC7

Standard deviation 1.937 1.1547 0.8137 0.72904 0.62981 0.48371 0.3004

Proportion of Variance 0.536 0.1905 0.0946 0.07593 0.05667 0.03343 0.0129

Cumulative Proportion 0.536 0.7265 0.8211 0.89701 0.95368 0.98710 1.0000

**Table SI.25 PCA loading for F1B1 germ line showing F3 corresponding broods for Ag NMs**

PC1 PC2 PC3 PC4 PC5 PC6 PC7

B.Actin -0.48589649 -0.009098843 0.38486383 0.3317285 0.4769169 -0.34204151 0.40151685

NADH -0.02824467 0.570035789 0.60168384 0.2032381 -0.2433440 0.02016676 -0.45968389

GST -0.11612286 0.602989201 -0.05424149 -0.5133791 -0.2002821 -0.03000450 0.56161026

DNA.poly -0.47565408 -0.180830659 -0.17257611 0.3531261 -0.7517841 -0.06648443 0.13028195

CAT -0.49648153 -0.069537531 -0.13109535 -0.5162623 0.0691516 -0.44533669 -0.51171397

MET -0.49605120 -0.103252712 0.12842208 -0.1803669 0.1434999 0.81928709 -0.04923461

HO1 -0.17554148 0.513014733 -0.65073306 0.4016657 0.2875197 0.08788834 -0.17555690

Importance of components:

PC1 PC2 PC3 PC4 PC5 PC6 PC7

Standard deviation 1.9592 1.5878 0.68455 0.31521 0.19966 0.15820 0.08647

Proportion of Variance 0.5484 0.3602 0.06694 0.01419 0.00569 0.00358 0.00107

Cumulative Proportion 0.5484 0.9085 0.97547 0.98966 0.99536 0.99893 1.00000

**Table SI.26 PCA loading for F1B1 germ line showing F4 corresponding broods for Ag NMs**

PC1 PC2 PC3 PC4 PC5 PC6 PC7

B.Actin 0.3960506 -0.28236642 -0.0212081 0.15208765 -0.38650024 -0.76758437 0.035560138

NADH -0.3783637 -0.37210572 0.4614731 0.03830936 0.45493052 -0.31323028 -0.445957634

GST -0.3727229 -0.36755524 -0.7102493 0.46311795 0.08292031 0.01268721 0.003417341

DNA.poly 0.2709924 -0.73193382 0.2867146 0.07408192 -0.06702038 0.46302657 0.290271097

CAT 0.4115433 -0.09881077 -0.2347327 -0.08678527 -0.11994759 0.26028722 -0.822253351

MET 0.4077782 -0.10720608 -0.3319631 -0.38894351 0.70337369 -0.16302228 0.198586073

HO1 -0.3898392 -0.29954274 -0.1861036 -0.77241611 -0.35095057 -0.06181231 0.007160771

Importance of components:

PC1 PC2 PC3 PC4 PC5 PC6 PC7

Standard deviation 2.4106 1.0273 0.29477 0.17019 0.12374 0.04823 0.01311

Proportion of Variance 0.8301 0.1508 0.01241 0.00414 0.00219 0.00033 0.00002

Cumulative Proportion 0.8301 0.9809 0.99332 0.99746 0.99964 0.99998 1.00000

**Table SI.27 PCA loading for F1B3 germ line showing F2 corresponding broods for Ag NMs**

PC1 PC2 PC3 PC4 PC5 PC6 PC7

B.Actin -0.4245206 0.1766650 -0.09207213 0.125545114 -0.41975841 0.36405228 0.67498271

NADH -0.3644681 -0.4766926 0.31407892 0.224585891 0.45371115 0.52367818 -0.10368297

GST -0.3712634 -0.4478663 -0.16484326 0.480496368 -0.33935381 -0.51158306 -0.16325120

DNA.poly -0.3393552 0.3698422 0.75017094 -0.007722345 0.09448517 -0.41312226 0.07510848

CAT -0.4191633 0.1867737 -0.02051390 -0.371101501 -0.40351225 0.27186644 -0.64385333

MET -0.3356117 0.5268277 -0.48909993 0.313794232 0.50159016 -0.05595004 -0.13194191

HO1 -0.3815937 -0.3029097 -0.25161052 -0.683139857 0.28186130 -0.28947902 0.26343911

Importance of components:

PC1 PC2 PC3 PC4 PC5 PC6 PC7

Standard deviation 2.2208 0.9391 0.71133 0.54428 0.4379 0.38017 0.21862

Proportion of Variance 0.7045 0.1260 0.07229 0.04232 0.0274 0.02065 0.00683

Cumulative Proportion 0.7045 0.8305 0.90281 0.94513 0.9725 0.99317 1.00000

**Table SI.28 PCA loading for F1B3 germ line showing F3 corresponding broods for Ag NMs**

PC1 PC2 PC3 PC4 PC5 PC6 PC7

B.Actin -0.3848210 -0.3702629 0.2761766 -0.31645418 -0.3518732 0.58917967 0.25971899

NADH -0.3861801 0.3920446 0.0745134 -0.46573940 0.1746350 -0.41613413 0.52061075

GST -0.3897834 0.3860516 0.1880424 -0.18434025 0.3435947 0.29359523 -0.65225481

DNA.poly -0.3433314 -0.2958301 -0.8548255 -0.13040994 0.2026534 0.06141156 -0.04511078

CAT -0.4033397 -0.2594684 0.1115843 0.01436862 -0.4955832 -0.58654393 -0.40951098

MET -0.3774163 -0.3680614 0.3074919 0.54292175 0.5385489 -0.09061378 0.18581373

HO1 -0.3575533 0.5194825 -0.2123331 0.58050409 -0.3884900 0.19361177 0.17836252

Importance of components:

PC1 PC2 PC3 PC4 PC5 PC6 PC7

Standard deviation 2.3556 0.8712 0.61158 0.40179 0.27831 0.22845 0.16422

Proportion of Variance 0.7927 0.1084 0.05343 0.02306 0.01106 0.00746 0.00385

Cumulative Proportion 0.7927 0.9011 0.95457 0.97763 0.98869 0.99615 1.00000

**Table SI.29 PCA loading for F1B3 germ line showing F4 corresponding broods for Ag NMs**

| PC1 PC2 PC3 PC4 PC5 PC6 PC7  B.Actin 0.4443104 -0.2350046 -0.0807952 0.70981403 -0.17713896 -0.18067009 0.4159055  NADH 0.2078046 0.5914904 0.1033843 -0.26131437 0.00905001 0.23922388 0.6860561  GST 0.2588493 0.5113861 -0.4761651 0.18941102 -0.37468626 0.24576944 -0.4561569  DNA.poly 0.4727267 -0.1989112 0.1060548 0.05443629 0.56913772 0.60382921 -0.1850012  CAT 0.4326922 -0.1845838 0.5581224 -0.30512612 -0.58555349 0.02840056 -0.1744255  MET 0.3230942 -0.3764776 -0.6453804 -0.53946713 -0.03240797 -0.13370956 0.1655462  HO1 0.4210934 0.3435462 0.1295626 -0.07008762 0.40038628 -0.68295907 -0.2370979  Importance of components:  PC1 PC2 PC3 PC4 PC5 PC6 PC7  Standard deviation 1.8989 1.4947 0.72904 0.52888 0.40085 0.35020 0.25608  Proportion of Variance 0.5151 0.3191 0.07593 0.03996 0.02295 0.01752 0.00937  Cumulative Proportion 0.5151 0.8343 0.91020 0.95016 0.97311 0.99063 1.00000 |
| --- |

**Table SI.30 PCA loading for F1B5 germ line showing F2 corresponding broods for Ag NMs**

PC1 PC2 PC3 PC4 PC5 PC6 PC7

B.Actin -0.4060406 0.2126590 -0.20903017 0.52855713 -0.06040546 0.54876453 0.40255390

NADH -0.3631384 -0.4206940 -0.49056425 -0.22137636 0.49394988 0.21822942 -0.33147503

GST -0.3771592 -0.4266185 -0.15131796 -0.33226525 -0.60902689 -0.18880456 0.36863177

DNA.poly -0.3542570 0.4633904 0.36067344 -0.64993298 0.06791726 0.32017189 0.01225947

CAT -0.4076348 0.2736135 -0.06567711 0.23194375 -0.41627566 -0.18703994 -0.70184666

MET -0.4086757 0.2628126 -0.04761773 0.09427335 0.42815394 -0.69006105 0.30538291

HO1 -0.3198944 -0.4869536 0.74571423 0.27590113 0.14225673 0.04368094 -0.07866092

Importance of components:

PC1 PC2 PC3 PC4 PC5 PC6 PC7

Standard deviation 2.3137 1.0046 0.60028 0.37746 0.26516 0.19947 0.15686

Proportion of Variance 0.7648 0.1442 0.05148 0.02035 0.01004 0.00568 0.00352

Cumulative Proportion 0.7648 0.9089 0.96040 0.98076 0.99080 0.99648 1.00000

**Table SI.31 PCA loading for F1B5 germ line showing F3 corresponding broods for Ag NMs**

PC1 PC2 PC3 PC4 PC5 PC6 PC7

B.Actin -0.2586613 -0.4721313 -0.76176900 -0.2544850 -0.1084744 -0.21480263 0.08499959

NADH -0.3991645 -0.2965404 0.42210924 0.3250589 -0.5053800 -0.41040136 0.21225867

GST -0.4714943 -0.1634728 -0.03691955 0.2433166 -0.1391608 0.63544877 -0.51695538

DNA.poly -0.2343118 0.5868088 -0.03167850 -0.5331919 -0.5509634 -0.02716623 -0.10562949

CAT -0.4262874 0.3439631 -0.17410828 0.2233195 0.1918869 0.29238102 0.70531976

MET -0.4206093 0.3378712 -0.09189903 0.2013471 0.4534700 -0.53829694 -0.40564282

HO1 -0.3713759 -0.2926096 0.44766737 -0.6290192 0.4091972 0.07514757 0.08543305

Importance of components:

PC1 PC2 PC3 PC4 PC5 PC6 PC7

Standard deviation 2.0125 1.3241 0.72977 0.60916 0.47200 0.22665 0.13685

Proportion of Variance 0.5786 0.2505 0.07608 0.05301 0.03183 0.00734 0.00268

Cumulative Proportion 0.5786 0.8291 0.90515 0.95816 0.98999 0.99732 1.00000

**Table SI.32 PCA loading for F1B5 germ line showing F4 corresponding broods for Ag NMs**

PC1 PC2 PC3 PC4 PC5 PC6 PC7

B.Actin -0.4386580 -0.2368711 0.07550027 -0.03600739 0.2535545 0.8208731 0.07969889

NADH -0.2684082 0.5399970 0.56313768 -0.49524392 0.2327410 -0.1271700 -0.06024249

GST -0.3018749 0.4022871 -0.81233584 -0.24570049 0.1542844 -0.0331550 0.04330644

DNA.poly -0.4139202 -0.3037325 0.04359136 0.33397658 0.5975414 -0.4963454 0.13986243

CAT -0.4242147 -0.3127167 -0.03887222 -0.16734823 -0.3055621 -0.1526204 -0.75899437

MET -0.4483200 -0.1441054 0.06682264 -0.14664974 -0.5913313 -0.1704231 0.61119115

HO1 -0.3035274 0.5288184 0.09700320 0.72939445 -0.2400862 0.1009956 -0.13767679

Importance of components:

PC1 PC2 PC3 PC4 PC5 PC6 PC7

Standard deviation 2.1296 1.2400 0.69554 0.5137 0.33074 0.21376 0.1565

Proportion of Variance 0.6479 0.2197 0.06911 0.0377 0.01563 0.00653 0.0035

Cumulative Proportion 0.6479 0.8675 0.93665 0.9744 0.98998 0.99650 1.0000

| **Table SI.33: Two tailed t-test for unequal variance of growth compared to the control generations for Daphnia exposed to Ag NPs** | | | | | | | | |
| --- | --- | --- | --- | --- | --- | --- | --- | --- |
| **Generation and exposure type (exposed/recovery)** | **Day 3** | **Day 6** | **Day 9** | **Day 12** | **Day 15** | **Day 18** | **Day 21** | **Day 24** |
| PVP only | 0.009 | 0.064 | 0.045 | 0.002 | 0 | 0.001 | 0.003 | 0.014 |
| F0 Bulk Ag | 0.445 | 0.033 | 0.215 | 0.174 | 0.002 | 0.005 | 0.015 | 0.016 |
| F1B1 Bulk Ag | 0.497 | 5.2593E-05 | 0.522 | 0.907 | 0.401 | 0.936 | N/A | N/A |
| F1B3 Bulk Ag | 0.002 | 0.886 | 0.407 | 0.894 | 0.156 | 0.486 | 0.46 | 0.129 |
| F2 Bulk Ag | 0.001 | 0.77 | 0.194 | 0.273 | 0.625 | 0.022 | 0.514 | 0.238 |
| F3 Bulk Ag | N/A | N/A | N/A | N/A | N/A | N/A | N/A | N/A |
| F1B5 Bulk Ag | N/A | 0.0002 | N/A | N/A | N/A | N/A | N/A | N/A |
| F0 Uncoated Ag | 0.033 | 0.059 | 0 | 0.123 | 0 | 0 | 0 | 0.001 |
| F1B1 Uncoated Ag | 0.382 | N/A | 0.035 | 0.146 | 2.491E-05  0.318 | 0.0128 | N/A | N/A |
| F1B1 Uncoated Ag recovery | N/A | N/A | N/A | 0.139 |  | 0.002 | N/A | N/A |
| F1B3 Uncoated Ag | 0 | 0.148 | 0.795 | 0.653 | 0.009 | 0.001 | 0.001 | 0 |
| F2 Uncoated Ag | 0.022 | 0.08 | N/A | N/A | N/A | N/A | N/A | N/A |
| F3 Uncoated Ag | N/A | 0 | 0.05 | 0.501 | N/A | N/A | N/A | N/A |
| F1B3 Uncoated Ag recovery | 0.032 | 0.165 | 0.311 | 0.591 | 0.083 | 0.501 | 0 | 0.001 |
| F2 Uncoated Ag recovery | 0.001 | 0.429 | 0.601 | 0.44 | 0.051 | 0.343 | 0.437 | 0.001 |
| F3 Uncoated Ag recovery | N/A | 0.025 | 0.015 | N/A | N/A | N/A | N/A | N/A |
| F1B5 Uncoated Ag | N/A | 0.155 | 0.218 | 0.377 | 0.029 | 0.328 | 0.109 | N/A |
| F1B5 Uncoated Ag recovery | N/A | 0.02 | 0.657 | 0.711 | 0.798 | 0.195 | 0.0377 | N/A |
| F0 PVP Ag | 0.177 | 0.878 | 0.618 | 0.723 | 0.864 | 0.038 | 0.719 | 0.897 |
| F1B1 PVP Ag | 0.776 | 0.0008 | 0.915 | 0.032 | 0.042 | 0.224 | N/A | N/A |
| F1B1 PVP Ag recovery | 0.335 | 0.0006 | 0.908 | 0.726 | 0.13 | 0.134 | N/A | N/A |
| F1B3 PVP Ag | 0.997 | 0.086 | 0.051 | 0.678 | 0.497 | 0.022 | 0 | 0.004 |
| F1B3 PVP Ag recovery | 0 | 0.122 | 0.001 | 0.853 | 0.978 | 0.025 | 0.426 | 0.988 |
| F2 PVP Ag recovery | 0.806 | 0.004 | N/A | N/A | 0 | 0.009 | 0.05 | 0.001 |
| F3 PVP Ag recovery | N/A | 0 | 0 | N/A | N/A | 0.004 | N/A | N/A |
| N/A F1B5 PVP Ag | N/A | 0.545 | N/A | N/A | N/A | N/A | N/A | N/A |
| F1B5 PVP Ag recovery | N/A | 0.555 | N/A | 0.0003 | N/A | N/A | N/A | N/A |
| F0 Ag_2_S | 0.022 | 0.511 | 0.119 | 0.088 | 0.006 | 0.945 | 0.008 | 0.116 |
| F1B1 Ag_2_S | 0.976 | 0.003 | 0.969 | 0.456 | 0.016 | 0.361 | N/A | N/A |
| F1B1 Ag_2_S recovery | 0.013 | 0.003 | 0.786 | 0.974 | 0.004 | 0.023 | N/A | N/A |
| F1B3 Ag_2_S | 0.046 | 0.527 | 0.039 | 0.015 | 0.127 | 0.233 | 0.876 | 0.486 |
| F2 Ag_2_S | 0.771 | 0.783 | 0.052 | 0.019 | 0.007 | 0 | 0.001 | 0 |
| F3 Ag_2_S | N/A | 0.594 | N/A | 0.013 | 0.217 | 0.001 | N/A | N/A |
| F1B3 Ag_2_S recovery | 0.147 | 0.234 | 0.018 | 0.016 | 0 | 0.645 | 0.683 | 0.847 |
| F2 Ag_2_S recovery | 0.617 | 0.707 | 0.021 | 0.009 | 0.038 | 0.012 | 0.015 | 0.063 |
| F3 Ag_2_S recovery | N/A | 0.142 | 0.067 | 0.798 | 0.117 | 0.431 | N/A | N/A |
| F1B5 Ag_2_S | N/A | 0.077 | N/A | N/A | N/A | N/A | N/A | N/A |
| F1B5 Ag_2_S recovery | N/A | 0.334 | 0.001 | 1.88E-05 | 0.017 | 0.014 | 0.032 | 0.769 |
| F0 TiO_2_ Uncoated | 0 | 0.001 | 0.005 | 0.029 | 0.292 | 0.085 | 0.016 | 0.129 |
| F1B5 TiO2 Uncoated | N/A | 0.109 | 0.0004 | 0.045 | 0.067 | 0.413 | N/A | N/A |
| F1B5 TiO2 Uncoated recovery | N/A | 0.545 | 0.007 | 0.045 | 0.463 | 0.609 | N/A | N/A |
| F0 TiO_2_ PVP | 0 | 0.005 | 0 | 0.385 | 0.018 | 0.167 | 0.823 | 0.532 |
| F1B1 TiO_2_ PVP | 0.046 | 5.96954E-09 | 0.018 | 0.014 | 0.221 | N/A | N/A | N/A |
| F1B1 TiO_2_ PVP recovery | 0.629 | 0.001 | 0.0005 | 0.119 | 0.388 | N/A | N/A | N/A |
| F1B3 TiO_2_ PVP | 0.135 | 0.001 | 0 | 0.771 | 0.3 | 0.005 | 0.001 | 0.166 |
| F2 TiO_2_ PVP | 0.052 | 0 | 0.734 | 0.039 | 0.028 | 0.018 | 0.037 | 0.003 |
| F3 TiO_2_ PVP | 0.011 | 0.000 | 0.002 | 0.012 | 0.082 | 0.022 | 0.043 | 0.011 |
| F1B3 TiO_2_ PVP recovery | 0.228 | 0.047 | 0.109 | 0.444 | 0.094 | 0.014 | 0.007 | 0.424 |
| F2 TiO_2_ PVP recovery | 0.025 | 0 | 0.036 | 0.168 | 0.215 | 0.189 | 0.138 | 0.000 |
| F3 TiO_2_ PVP recovery | 0.012 | 0.168 | 0.089 | 0.043 | 0.017 | 0.228 | 0.014 | 0.002 |

**References**

ELLIS, L.-J. A., VALSAMI-JONES, E. & LYNCH, I. 2020. Exposure medium and particle ageing moderate the toxicological effects of nanomaterials to Daphnia magna over multiple generations: a case for standard test review? *Environmental Science: Nano*.

HECKMANN, L.-H., CONNON, R., HUTCHINSON, T. H., MAUND, S. J., SIBLY, R. M. & CALLAGHAN, A. 2006. Expression of target and reference genes in Daphnia magna exposed to ibuprofen. *BMC genomics,* 7**,** 175.

KILHAM, S. S., KREEGER, D. A., LYNN, S. G., GOULDEN, C. E. & HERRERA, L. 1998. COMBO: a defined freshwater culture medium for algae and zooplankton. *Hydrobiologia,* 377**,** 147-159.

KIM, K.-T., KLAINE, S. J. & KIM, S. D. 2014. Acute and chronic response of daphnia magna exposed to TiO 2 nanoparticles in agitation system. *Bulletin of environmental contamination and toxicology,* 93**,** 456-460.

KIM, K. T., KLAINE, S. J., CHO, J., KIM, S.-H. & KIM, S. D. 2010. Oxidative stress responses of Daphnia magna exposed to TiO 2 nanoparticles according to size fraction. *Science of the Total Environment,* 408**,** 2268-2272.

POYNTON, H. C., VARSHAVSKY, J. R., CHANG, B., CAVIGIOLIO, G., CHAN, S., HOLMAN, P. S., LOGUINOV, A. V., BAUER, D. J., KOMACHI, K. & THEIL, E. C. 2007. Daphnia magna ecotoxicogenomics provides mechanistic insights into metal toxicity. *Environmental science & technology,* 41**,** 1044-1050.

QIU, T., BOZICH, J., LOHSE, S., VARTANIAN, A., JACOB, L., MEYER, B., GUNSOLUS, I., NIEMUTH, N., MURPHY, C. & HAYNES, C. 2015. Gene expression as an indicator of the molecular response and toxicity in the bacterium Shewanella oneidensis and the water flea Daphnia magna exposed to functionalized gold nanoparticles. *Environmental Science: Nano,* 2**,** 615-629.

ZHU, X. C., YUNG CHEN, YONGSHENG 2010. Toxicity and bioaccumulation of TiO 2 nanoparticle aggregates in Daphnia magna. *Chemosphere,* 78**,** 209-215.
